# FIBCD1 Binds *Aspergillus fumigatus* and Regulates Lung Epithelial Response to Cell Wall Components

**DOI:** 10.3389/fimmu.2018.01967

**Published:** 2018-09-18

**Authors:** Christine Schoeler Jepsen, Lalit Kumar Dubey, Kimmie B. Colmorten, Jesper B. Moeller, Mark A. Hammond, Ole Nielsen, Anders Schlosser, Steven P. Templeton, Grith L. Sorensen, Uffe Holmskov

**Affiliations:** ^1^Cancer and Inflammation Research, Department of Molecular Medicine, University of Southern Denmark, Odense, Denmark; ^2^Global Health Institute, School of Life Sciences, École Polytechnique Fédérale de Lausanne, Lausanne, Switzerland; ^3^Jill Roberts Institute for Research in Inflammatory Bowel Disease, Weill Cornell Medicine, Cornell University, New York, NY, United States; ^4^Department of Clinical Pathology, Odense University Hospital, Odense, Denmark; ^5^Department of Microbiology and Immunology, Indiana University School of Medicine-Terre Haute, Terre Haute, IN, United States

**Keywords:** FIBCD1, *Aspergillus fumigatus*, human, A549, lung, inflammation, IL-8, epithelium

## Abstract

*Aspergillus fumigatus* (*A. fumigatus*) is a ubiquitous fungus of clinical importance associated with development of various pulmonary diseases and allergic hypersensitivity reactions. It is protected against environmental stress by a cell wall that contains polysaccharides such as chitin. We previously demonstrated that fibrinogen C domain-containing protein 1 (FIBCD1) is a membrane-bound protein that binds chitin through a conserved S1 binding site and is expressed in intestinal epithelium and salivary glands. Here, we further localized FIBCD1 protein expression at the surface of bronchial and alveolar human lung epithelium, observed recognition of *A. fumigat*us cell wall with S1 site-independent recognition. We observed FIBCD1-mediated suppression of IL-8 secretion, mucin production, and transcription of genes associated with airway inflammation and homeostasis in FIBCD1-transfected lung epithelial cells. These modulations were generally enforced by stimulation with *A. fumigatus* cell wall polysaccharides. In parallel, we demonstrated a FIBCD1-mediated modulation of IL-8 secretion induced by TLR2,−4, and −5. Collectively, our findings support FIBCD1 as a human lung epithelial pattern recognition receptor that recognizes the complex *A. fumigatus* cell wall polysaccharides and modulates the lung epithelial inflammatory response by suppressing inflammatory mediators and mucins.

## Introduction

*Aspergillus fumigatus* (*A. fumigatus*) is a ubiquitous, filamentous fungus with the ability to cause invasive and allergic pulmonary diseases that is partly attributed to its ability to circumvent host immune defenses at airway mucosal sites (1–4). Humans inhale several hundred *A. fumigatus* conidia every day and their small size make them easily aerosolized and capable of reaching the lung alveoli. In healthy, immune-competent hosts, inhaled *A. fumigatus* conidia are cleared by innate defense mechanisms including mucociliary transport mechanisms and phagocytic activity of leukocytes, primarily residential macrophages and neutrophils recruited by epithelial secretion of chemotactic factors such as IL-8. Additionally, epithelial secretion of opsonizing mediators such as ficolins and complement components support the activity of these mechanisms (5, 6).

A cell wall, mainly composed of polysaccharides and secretory antigens, protects *A. fumigatus* conidia and hyphae against environmental stress (7). The structural skeleton of the cell wall is composed of chitin and galactomannan covalently bound to β-1,3-glucan (8), which are polysaccharide structures absent in mammals. Therefore, they serve as pathogen-associated molecular patterns (PAMPs) recognized by pattern recognition receptors (PRRs) on mammalian cells (9). The *A. fumigatus* cell wall is continuously remodeled during morphogenesis from inhaled, resting conidia to fully-grown hyphae (10). In resting conidia, the cell wall is protected by an additional layer of hydrophobic proteins and pigments, not produced in germ tubes and hyphae. This layer shields *A. fumigatus* cell wall PAMPs, which makes the resting conidia immunologically inert (9). During the first stages of germination, cell wall polysaccharides are hydrolyzed and *de novo* synthesis of cell wall components, e.g. chitin and β-1,3-glucan, is initiated (10). This causes the conidia to swell, disintegrating the outer layer and exposing cell wall PAMPs to host PRRs (9). Various PRRs are involved in the initiation, control, and resolution of the anti-*A. fumigatus* inflammatory response (11), including Toll-Like Receptors (TLRs) (5, 6) and the β-1,3-glucan receptor Dectin-1 (12). Several studies report a TLR-regulated anti-*A. fumigatus* response dependent on the fungus' morphotype (5, 6, 13). However, the underlying mechanisms remain unknown and may involve interplay between other PRRs that recognize *A. fumigatus*-associated PAMPs.

We have previously identified fibrinogen C domain-containing protein 1 (FIBCD1) as the first membrane-bound protein of the fibrinogen-related domain (FReD) superfamily (14). FIBCD1 is expressed as a tetramer on the apical surface of small and large intestinal epithelium and is composed of a cytoplasmic tail with three potential phosphorylation sites, a transmembrane region, and an ectodomain containing a coiled-coil region, a polycationic region, and a FReD (14). FIBCD1 exhibits calcium-dependent binding of acetylated structures including crab shell chitin through a conserved S1 binding site in FReD and facilitates endocytosis of bound ligands (14, 15). The crystal structure of FIBCD1-FReD (16) is homologous to FReD superfamily members involved in immune regulation, including ficolin-1 and −2 (17, 18). Both ficolin-1 and −2 recognize *A. fumigatus*-associated PAMPs (19, 20) and binds directly or indirectly to resting *A. fumigatus* conidia (21).

The aim of this study is to investigate the potential role of FIBCD1 in the immune response against *A. fumigatus*. We hypothesize that FIBCD1 is expressed in human lung epithelium and interacts with *A. fumigatus* by binding fungal cell wall chitin in the S1 binding site of FIBCD1-FReD. We hypothesize that interaction between FIBCD1 and ligand(s) modulates TLR- and lung epithelial cell-mediated inflammatory response to fungal cell wall components.

## Methods and materials

### Buffers and reagents

PBS: 140 mM NaCl, 3 mM KCl, 8 mM Na_2_HPO_4_, 1.5 mM KH_2_PO_4_. TBS: 10 mM Tris-base, 140 mM NaCl. SDS-PAGE sample buffer: 374 mM Tris-base, 40% (v/v) glycerol, 3 mM bromophenol blue, 8% (w/v) SDS. Acetylated BSA (acBSA), BSA, citric acid, EDTA, glycine, glycerol, silver nitrate (AgNO_3_), SDS, sodium azide (NaN_3_), Tris-base, Triton X-100, H_2_SO_4_, iodoacetamide (IAA), DTT, CaCl_2_, sodium acetate, NaHCO_3_, sterile PBS, curdlan (β-1,3-glucan, from *Alcaligenes faecalis*), RNase ZAP®, M-MLV reverse transcriptase, and oligo-dT primers were ordered from Sigma-Aldrich Co., St Louis, MO, United States. Alexa Fluor 488, Alexa Fluor 633-labeled wheat germ agglutinin (WGA), Gateway Technology® pDONR^TM^-221 vector, 3-(N-morpholino)-propanesulfonic acid (MOPS) running buffer, RPMI media, FBS, L-glutamine, penicillin/streptomycin, hygromycin B, trypsin/EDTA, Dulbecco's PBS (DPBS), DMSO, TRIzol reagent, and RNaseOUT^TM^ recombinant ribonuclease inhibitor were ordered from Invitrogen^TM^, Thermo Fisher Scientific Inc., Waltham, MA, United states. Sabouraud dextrose (SD) agar, SD broth media, and 40-μm cell strainer were ordered from BD Biosciences, Franklin Lakes, NJ, United States. Acetic acid, bromophenol blue, formaldehyde, KCl, KH_2_PO_4_, NaCl, Na_2_HPO_4_, Na_2_CO_3_, NaOH, Tween 20, and mira cloth were ordered from Merck KGaA, Darmstadt, Germany. Four to twelve percent of polyacrylamide gradient gels, Precision Plus Protein^TM^ KaleidoscopeTM Standards, and non-fat dry milk were ordered from Bio-Rad Laboratories, Inc., Hercules, CA, United States. Chitin beads were ordered from New England Biolabs Inc., Ipswich, Massachusetts, USA, and Dectin-1Fc was kindly provided by Prof Gordon Brown, Aberdeen University. HRP-conjugated rabbit anti-mouse Ig (#P0260), FITC-conjugated goat anti-mouse antibody (#F0479), and HRP-conjugated goat anti-rabbit Ig (#P0448) were ordered from Dako, Glostrup, Denmark. D-galacto-D-mannan (from Ceratonia Siliqua) and mouse monoclonal anti-GAPDH antibody (#sc32233) were ordered from Santa Cruz Biotechnologies, Inc., Dallas, TX, United States.

The anti-mucin (MUC) antibodies rabbit monoclonal anti-MUC-1 antibody (#ab45167), rabbit polyclonal anti-MUC-13 antibody (#ab65109), and mouse monoclonal anti-MUC-5AC antibody (#ab11335) were ordered from Abcam plc, Cambridge, United Kingdom. Protein G column, polyvinylidene difluoride membrane, filter paper, and ECL kit were ordered from GE Healthcare, Little Chalfont, United Kingdom. Ambion® nuclease-free water, custom TaqMan® array 96-well plates, and 2 X TaqMan® fast advanced master mix were ordered from Thermo Fisher Scientific Inc., Waltham, MA, United states. The human TLR agonist kit was ordered from InvivoGen, San Diego, CA, United States. Alphazyme was ordered from PAA Laboratories GmbH, Pasching, Austria. O-phenylendiamid was ordered from Kem-En-Tec Diagnostics A/S, Taastrup, Denmark. Human CXCL8/IL-8 DuoSet kit was ordered from R&D Systems, Inc., Minneapolis, MN, United states. Limulus amebocyte lysate assay (QCL-1000™) was ordered from Lonza Group Ltd., Basel, Switzerland. Protease inhibitor cocktail tablets were ordered from Roche Diagnostics, Basel, Switzerland. Commercial cDNA library and FIBCD1 PerfectProbe™ assay were ordered from Primerdesign Ltd., United Kingdom. JetPEI transfection reagents were ordered from Polyplus transfection SA, Illkirch, France. Monoclonal anti-ovalbumin antibody was ordered from SSI, Copenhagen, Denmark. The murine Sp2/mIl-6 (CRL-2016) myeloma cells and human lung carcinoma type II epithelial-like A549 cell line were purchased from the American type culture collection (Rockville, MD, USA).

### Real time analysis of FIBCD1 expression in human tissues

The relative tissue distribution of human FIBCD1 mRNA was quantitated using a commercial cDNA library and a custom made FIBCD1 PerfectProbe™ assay with the primer sequences 5′-CACCGTGGCTGACTATTCC-3′ and 5′-TTCTCTGAATGGTCGCTGTC-3′. Analysis was performed in triplicates using the cycling conditions: 95°C for 10 min, followed by 50 cycles of 95°C for 15 s, 60°C for 30 s and 72°C for 15 s. The study was performed on a 7500 Real-time PCR system (Applied Biosystems®, Thermo Fisher Scientific Inc., Waltham, MA, USA) using 18S RNA for normalization.

### Expression and purification of recombinant FIBCD1-FReD (rfibcd1-FReD)

rFIBCD1-FReD was expressed in insect cells and purified on N-acetylated immobilized resin as described previously (15).

### Production of monoclonal Anti-FIBCD1 antibodies

Mouse monoclonal anti-FIBCD1 antibodies HG-HYB-12-2,−12-5, and −12-6 were produced as previously described (14). Mouse monoclonal anti-FIBCD1-FReD antibody HYB-11-14-25 was produced by immunization of *Fibcd1*^−/−^ C57BL6/n mice. The mice were immunized twice with 20 μg of rFIBCD1-FReD using GERBU as adjuvant with more than 2 weeks between each immunization. The mice were boosted once by an intra-peritoneal injection with 20 μg rFIBCD1-FReD without adjuvant 3 days before isolation of B cells. Hybridoma cells were produced by fusion between isolated B cells and myeloma cells and adapted for serum-free media. Secreted antibodies were purified using a protein G column on a fast protein liquid chromatography apparatus (ÄKTA, GE Healthcare, Little Chalfont, United Kingdom). These experiments were performed under license from the National Animal Experiments Inspectorate (reference no. 2012–15–2934–00076).

### Immunohistochemical analysis of FIBCD1 in human lung tissue

Human tissues were obtained from the tissue bank at the Department of Pathology, Odense University Hospital (Odense, Denmark). The tissues were fixed in 4% formalin in PBS for 24 h and then conventionally dehydrated and embedded in paraffin. A biotin-streptavidin immunoperoxidase technique was used on paraffin sections. Paraffin sections were pre-treated in TEG buffer (10 mmol/L Tris, 0.5 mmol/L EGTA, pH 9) in a microwave oven for three 5 min periods at 650 W. The sections were left in TEG buffer for 15 min, washed in TBS, pre-incubated with 2% (w/v) BSA in TBS for 10 min, and incubated for 30 min with the mouse anti-human FIBCD1 (HG-HYB-12-2, 0.5 mg/mL) in TBS containing 15% (w/v) BSA and otherwise processed as described by Madsen et al. (22). The specificity of the immuno-staining was verified by replacing the primary antibody with a non-specific antibody. The local ethical committee in Odense approved the use of human tissue samples (ref. no: VF20050070).

### Fungal strain and growth conditions

*A. fumigatus* conidia (101355, Centraalbureau von Schimmelcultures, Utrecht, Netherlands) were cultivated on SD agar plates at 37°C for 5 days and harvested using PBS/0.5% Tween, washed, and used immediately or stored at 4°C. *A. fumigatus* germination was obtained by inoculating SD broth media with freshly-harvested conidia (less than a week after harvest) and incubating at 37°C. For production of a 1-week-culture, 10^4^ freshly-harvested conidia were added per mL media in an Erlenmeyer flask containing 1:4 volume media and incubating at 37°C, 100–140 rpm for 7 days.

### Alexa fluor 488-labeling of rFIBCD1-FReD

Purified protein (2 mg/mL) was labeled with Alexa Fluor 488 in a 1:9 molar ratio at RT for 1–2 h according to manufacturer's recommendations followed by extensive dialysis against PBS to remove excess dye. Activity of labeled rFIBCD1-FReD was tested by binding to acBSA. Labeled protein was stored at 4°C until used.

### Staining of *A. fumigatus*

Staining of *A. fumigatus* was performed by a modification of previously described methods (23) and imaged using an Olympus IX71 fluorescence microscope equipped with four laser optics and F-view fluorescence CCD camera (Olympus Corporation, Tokyo, Japan). All images were acquired and processed using Cell^F^ soft imaging software (Olympus Corporation, Tokyo, Japan).

### Preparation of *A. fumigatus* alkali-insoluble fraction (AIF)

*A. fumigatus* cell wall component suspension (i.e., alkali-insoluble fraction) was prepared as previously described with minor modifications (8). Briefly, mycelium was harvested from one-week-culture by filtration through mira cloth, washed with distilled PBS, and subjected to three hot alkali treatments (distilled 1 M NaOH, 65°C heat bath, 30 min), each followed by five washes with distilled water and one with distilled TBS at 10,000 × g for 10 min. The product was grinded with mortar and pestle, washed with sterile PBS, forced through a 40 μm cell strainer, pH adjusted to ~7, added 0.05% NaN_3_, and the final product stored at 4°C. AIF was tested for endotoxin contamination using a limulus amebocyte lysate assay revealing an endotoxin level below 0.25 endotoxin units per mL. The absence of protein was confirmed by boiling AIF 1:2 in SDS-PAGE sample buffer on a 99°C heating block for 1 min and separating eluted protein on a 4–12% polyacrylamide gradient gel using MOPS running buffer and subsequently silver staining the gel as described in the following section. The concentration was determined by vacuum drying using a MAXI-dry vacuum centrifuge (Heto-Holten A/S, Alleroed, Denmark) at 55°C, 1,300 rpm.

### Pull down assay

For protein staining, 2 mg of AIF was washed three times with TBS/0.05% Tween/5 mM CaCl_2_ and incubated at 4°C overnight with 20 μg/mL Dectin-1Fc, recombinant mannan-binding lectin (rMBL), WGA, and rFIBCD1-FReD in a total volume of 1 mL. Pull down was performed by centrifugation at 4°C, 10,000 × g for 5 min. The insoluble pellets were washed three times with TBS/0.05% Tween/5 mM CaCl_2_ and bound protein was eluted by boiling the pellets in SDS-PAGE sample buffer for 1 min. For Western blotting, 2 mg AIF and β-1,3-glucan and 200 μL chitin beads were washed three times with TBS/0.05% Tween and incubated at 4°C overnight with 5 μg/mL rFIBCD1-FReD in a total volume of 1 mL TBS/0.05% Tween supplemented with 5 mM CaCl_2_, 10 mM EDTA, or 100 mM sodium acetate. Pull down was performed by centrifugation at 5,000 rpm (Minispin, Eppendorf AG, Hamburg, Germany) for 5 min. The insoluble pellets were washed three times with TBS/0.05% Tween supplemented with 5 mM CaCl_2_, 10 mM EDTA, or 100 mM sodium acetate and bound protein was eluted by boiling the pellets in SDS-PAGE sample buffer on a 99°C heating block for 1 min. Pull down of mutant FIBCD1-FReD (A432V) by AIF and chitin beads was performed as previously described (15). Eluted protein was separated by SDS-PAGE on a 4–12% polyacrylamide gradient gel using MOPS running buffer and analyzed by silver staining or Western blotting as described in the following section.

### Silver staining

Silver staining of proteins separated by SDS-PAGE was performed as previously described (24) with some modifications (Supplementary Datasheet 2).

### Generation of A549 cells expressing full length of FIBCD1

To generate A549 cells stably expressing full length FIBCD1, the cells were transfected with the expression vector pDEST-FIBCD1 using JetPEI transfection reagents according to manufacturer's instructions. The pDEST-FIBCD1 vector was constructed using Gateway Technology® and keeping to the recommendations of the manufacturer using cDNA containing the open reading frame of FIBCD1, donor vector pDONR^TM^-221, and destination vector pDEST. A corresponding sham-transfected isogenic control cell type was constructed using a pDEST-sham vector. Cells were selected for stable integration of vectors using 500 μg/mL of hygromycin B. Successful expression of FIBCD1 protein was confirmed by Western blotting of cell lysates (Supplementary Datasheet 2) and surface expression of FIBCD1 was confirmed by flow cytometry. Unless otherwise stated, A549 cells were maintained in serial passages at 37°C, 5% CO_2_ humidity in RPMI media supplemented with 10% FBS, 2 mM L-glutamine, 250 μg/mL hygromycin B, 50 U/mL penicillin, and 50 μg/mL streptomycin. When confluent, cells were subcultured by washing twice in sterile DPBS, detaching with 1 mL 0.5% trypsin/EDTA/sterile DPBS, and diluted using fresh complete media.

### Flow cytometry

A549 cells were harvested by incubation with alphazyme and centrifugation at 350 × g for 5 min. Cells were suspended in media, counted using a haemocytometer, portioned out in minisorp tubes (10^6^ cells/tube), pelleted by centrifugation, and supernatant removed. The cells were incubated on ice for 2 h with 0.1 mg/mL monoclonal mouse anti-FIBCD1 antibody HG-HYB-12-5 in PBS/0.5% BSA diluted 1:2 in 100 μL cell suspension. Monoclonal anti-ovalbumin antibody was used as an isotype control. Then, the cells were washed three times and incubated in darkness on ice for 1 h with FITC-conjugated goat anti-mouse antibody diluted 1:10 in PBS/0.5% BSA and 1:2 in 100 μL cell suspension. Finally, the cells were washed three times, diluted in 1.5 mL PBS/0.5% BSA, and analyzed on a Benson Dickinson FACS Calibur (BD Biosciences, Franklin Lakes, NJ, United States) using CELL Quest^TM^ (BD Biosciences, Franklin Lakes, NJ, United States) and FlowJo 8.8.6 software (FlowJo, LLC, Ashland, OR, United States).

### Cell culture stimulation conditions, relative gene expression, and protein expression

For stimulations of transfected cells, A549 cells transfected with sham and FIBCD1, respectively, were harvested by incubation with 0.5% trypsin/EDTA in DPBS and centrifugation at 350 × g for 5 min. Cells were suspended in media, counted using a haemocytometer, and seeded at a density of 10^6^ cells/well/2 mL media in 6-well culture plates or 250,000 cells/well/0.5 mL media in 24-well culture plates (80% confluence). After ~8 h, the adherent cells were washed with sterile DPBS, added 2 or 0.5 mL serum-free media, and incubated overnight. Then, cells were stimulated by removing the media and adding 2 mL of fresh serum-free media containing 100 μL conidia, AIF, β-1,3-glucan, chitin, galactomannan, or acBSA (ligand control) diluted in sterile DPBS or 0.5 mL of fresh serum-free media containing 22.2 μL human TLR agonist diluted in endotoxin-free water to each well and incubating 0, 4, and 8 h. All stimulations were performed as technical duplicates and biological triplicates.

At each time point, culture supernatant was removed from the cells and used for detection of secreted IL-8 by use of the human CXCL8/IL-8 DuoSet kit and keeping to the recommendations of the manufacturer. For RNA analysis, the cells were washed with DPBS, added TRIzol Reagent, and stored at −20°C. Total RNA was isolated from cells stimulated 8 h with 500 μg/mL AIF, β-1,3-glucan, or chitin, and cells incubated with DPBS for 8 h as a reference. TRIzol Reagent from technical duplicates of stimulated cells was pooled together, while five biological replicates from DPBS-incubated cells were pooled together to three independent samples. Total RNA was isolated from TRIzol Reagent according to the recommendations of the manufacturer in a fume cupboard treated with RNase ZAP®. cDNA synthesis by M-MLV reverse transcriptase and oligo-dT primers was subsequently performed in keep with manufacturer's instructions. The RNA concentration and purity (260/280) was determined by NanoDrop® ND-1000 spectrophotometry (Thermo Fisher Scientific Inc., Waltham, MA, United states). Then, quantitative PCR (qPCR) was performed targeting 21 different genes involved in immunological response and barrier function (CCL2, CCL5, CCL20, CXCL2, CSF2RA, TNF, TSLP, IL1B, IL6 IL8, IL10, IL12B, IL13, IL25, IL33, MUC1, MUC13, MUC5AC, TJP1, OCLN, ICAM1) and two housekeeping genes (GAPDH, TBP) for normalization. Custom TaqMan® Array 96-well Plates containing dried assay of these genes were added 10 μL cDNA diluted 1:2 in 2 X TaqMan® fast advanced master mix as single copies and qPCR was performed according to the recommendations of the manufacturer using a StepOnePlus™ Real-Time PCR System (Applied Biosystems®, Thermo Fisher Scientific Inc., Waltham, MA, USA) and StepOne^TM^ v2.1 software (Applied Biosystems®, Thermo Fisher Scientific Inc., Waltham, MA, USA). A no-template control was included to exclude DNA contamination. Relative expression of the genes was calculated by qBase plus software (Biogazelle, Gent, Belgium), which uses threshold cycle values of target genes and reference genes to determine calibrated normalized relative quantities (CNRQs). For protein analysis, cells were washed twice with DPBS, added 11 μL/cm^2^ RIPA buffer/protease inhibitors (1 tablet per 50 mL)/phosphatase inhibitors (1 tablet per 10 mL), and incubated on a shaker at 4°C for 60 min. Cellular debris were sedimented by centrifugation at 4°C, 10,000 rpm (Sigma 1–16 k refrigerated centrifuge, Sigma-Aldrich Co., St Louis, MO, United States) for 5 min and supernatants were stored at −20°C. Protein concentrations were determined by Bradford in accordance with the recommendations of the manufacturer and samples were prepared for SDS-PAGE by boiling three parts cell lysate with one part SDS-PAGE sample buffer on a 99°C heating block for 1 min. The samples were alkylated by adding one tenth 1.4 M IAA, separated on a 4–12% polyacrylamide gradient gel (19 μg protein per well) using MOPS running buffer, and analyzed by Western blotting as described in the following section.

### Western blotting

Proteins separated by SDS-PAGE were transferred onto a polyvinylidene difluoride membrane by semidry electroblotting (1.2 mA/cm^2^ for 1 h or 0.2 mA/cm^2^ overnight) using transfer buffer (75 mM Tris-base/39 mM glycine/0.037% (w/v) SDS/20% ethanol) and blocked with TBS/0.5 M NaCl/0.1% Tween/5% non-fat dry milk at 4°C for several hours. FIBCD1 was detected by probing the blot with 2 μg/mL mouse monoclonal anti-FIBCD1 antibody HG-HYB-12-2 or mouse monoclonal anti-FIBCD1-FReD antibody HYB-11-14-25 diluted in TBS/0.5 M NaCl/0.1 % Tween/2.5% non-fat dry milk at 4°C overnight. Mucins were detected by probing the blot with 0.22 μg/mL rabbit monoclonal anti-MUC-1 antibody, 2 μg/mL rabbit polyclonal anti-MUC-13 antibody, or 6.6 μg/mL mouse monoclonal anti-MUC-5AC antibody in 20 mL TBS/0.5 M NaCl/0.1% Tween/2.5% non-fat dry milk at 4°C overnight. Mouse monoclonal anti-GAPDH antibody (0.01 μg/mL) was used as control. Excess antibody was removed by extensive washing with TBS/0.5 M NaCl/0.1% Tween and the blot was incubated 1 h with either HRP-conjugated rabbit anti-mouse antibody diluted 1:10,000 or HRP-conjugated goat anti-rabbit antibody diluted 1:20,000 in TBS/0.5 M NaCl/0.1% Tween. The blot was washed, developed using ECL standard method, detected by Fusion Fx7 (Vilber Lourmat, Collégien, France), and depicted using FUSION-CAPT version 15.18 software (Vilber Lourmat, Collégien, France). Precision Plus Protein^TM^ Kaleidoscope^TM^ Standards were used as size markers. Between incubations with anti-mucin and -GAPDH antibodies, the membrane was stripped and blocked. Stripping was achieved by washing once with TBS/0.5 M NaCl/0.1% Tween, boiling in deionized water for 10 min, and washing twice with TBS/0.5 M NaCl/0.1% Tween.

### Expression of results and statistics

Unless otherwise stated, data are expressed as mean ± SEM and differences were considered to be statistically significant when *p* < 0.05. Log2-transformed IL-8 secretion data was analyzed by one- or two-way ANOVA with Tukey's *post-hoc* tests depending on the number of independent variables using Prism software (version 6.0d, Graphpad, San Diego, CA, USA). Multilevel mixed-effects linear regression models were used to determine whether changed RNA expression of various genes was associated with stimulant, genotype, or a combined effect of these variables using the XTMIXED function of STATA13 (STATA Corp, College Station, TX, USA). Genes were analyzed separately using multilevel mixed-effects linear regression models to compensate for random effects. Log2 (CNRQ) was outcome, while stimulant, genotype, and interaction between these were fixed effects, and biological triplicate and culture plate variation were random effects (Table S1). Final models were evaluated for normality by prediction of residuals and assessment of their normal distribution by graphic (qq-plot, histogram, and box plot) and numeric methods (Skewness/Kurtosis and Shapiro-Wilk tests). *P*-values were extracted from the models using the LINCOM function.

## Results

### FIBCD1 is expressed on the apical surface of human bronchial and alveolar epithelial cells

**Figure 1:**
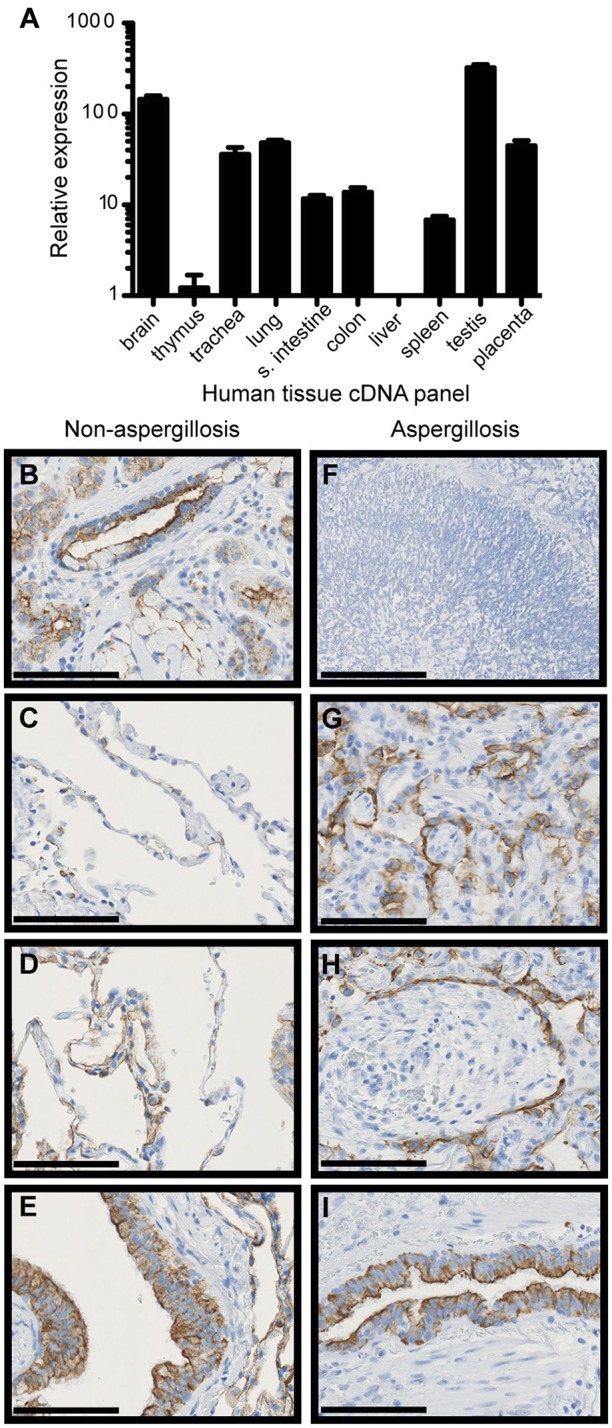
FIBCD1 RNA is expressed in various human tissues and FIBCD1 protein is expressed on the apical site of lung epithelium and within submucosal glands. **(A)** Relative expression of FIBCD1 mRNA in various human tissues relative to expression in thymus. Technical triplicates. **(B–E)** IHC staining of FIBCD1 in non-cancerous lung tissue from a 68-year-old female patient with adenocarcinoma (scale bars: 100 μm, magnification: 40X). FIBCD1 was detected in submucosal glands **(B)**, non-inflamed alveoli **(C)**, inflamed alveoli **(D)**, and inflamed bronchioles **(E)**. **(G–I)** IHC staining of FIBCD1 in lung tissue from a 63-year-old male patient with *A. fumigatus* infiltration (scale bars: 100 μm, magnification: 40X). Formation of aspergilloma was observed (**F**, scale bar: 200 μm, magnification: 20X) and FIBCD1 was detected in alveoli with atelectasis **(G)** and pneumonia **(H)** and in bronchioles **(I)**.

We have previously shown that FIBCD1 is expressed apically by epithelial cells of mucous membranes, i.e. the small and large intestine and salivary glands (14), and therefore hypothesized that it is also expressed in the lung mucosal membrane. First, we determined FIBCD1 mRNA expression in a series of human tissues (Figure 1A). The highest expressional levels were found in the respiratory tract (lung and trachea), gastrointestinal tract (colon and small intestine), testis, placenta, and brain. Then, we performed an immunohistochemical (IHC) analysis of human, non-cancerous lung tissue (control) from patients with lung cancer (Figures 1B–E) and from patients with pulmonary *A. fumigatus* infection (Figures 1F–I). FIBCD1 immunostaining was detected in submucosal glands, alveoli, and bronchioles with more intense staining coincident with areas of inflammation (Figures 1G,H). Bronchiolar FIBCD1 was restricted to the apical surface of ciliated epithelial cells (Figures 1E,I) similar to the expression observed in the gastrointestinal tract (14). Similar results were observed by von Huth et al. (25) in non-malignant, non-inflammatory, and histologically normal, human tissues. Thus, FIBCD1 is expressed apically in the lung mucosal membrane and seemingly increased in areas of inflammation.

### FIBCD1 binds to *A. fumigatus* dependent on cell wall PAMP availability

**Figure 2:**
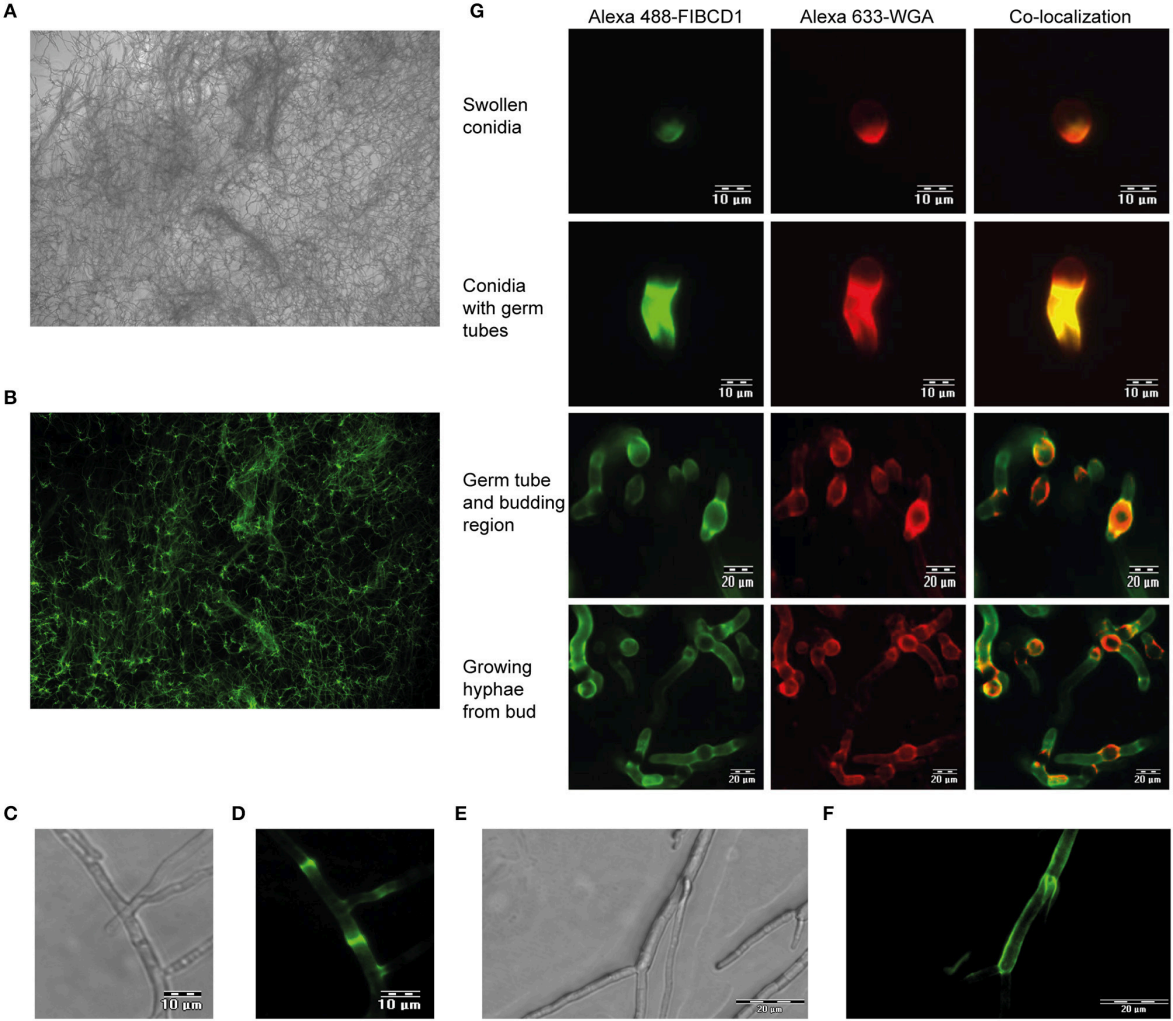
FIBCD1 recognizes chitin-rich regions of *A. fumigatus* during different life stages. Bright field **(A,C,E)** and fluorescence **(B,D,F,G)** microscopy of *A. fumigatus*. **(A,B)** A total of 10^7^ *A. fumigatus* conidia/mL was grown in SD medium to form fungal hyphae followed by staining with Alexa 488-labeled FIBCD1 as described in materials and methods. A clear recognition of fungal hyphae by FIBCD1 was observed. Image: 10X objective. **(C–F)** 5·10^6^ *A. fumigatus* conidia/mL were grown in SD broth medium and stained with Alexa 488-labeled FIBCD1. rhFIBCD1 recognized the chitin-rich *A. fumigatus* septum of fully-grown hyphae. Image 40X and 20X objectives. **(G)** 10^4^ *A. fumigatus* conidia/mL was grown in SD medium for various time points (6, 8, and 12 h) at 37°C and stained with Alexa 633-labeled WGA and Alexa 488-labeled FIBCD1. FIBCD1 failed to bind resting conidia (Figure S1). We observed co-localization of regions recognized by Alexa 488-labeled FIBCD1 and Alexa 633-labeled WGA.

We next examined if FIBCD1 is capable of recognizing *A. fumigatus* hyphae. Fluorescence microscopy revealed FIBCD1 recognition of fully-grown hyphae (Figures 2A,B), including chitin-rich septum regions (Figures 2C,D) and mycelial frame of budding hyphae (Figures 2E,F). We further examined co-localization between the chitin-binding fluorescent-labeled WGA and FIBCD1 in different morphological stages. Co-localization was observed in all stages characterized by exposed cell wall polysaccharides including swollen conidia, germ tubes, budding regions, and growing hyphae (Figure 2G), while no binding of FIBCD1 observed on resting conidia (Figure S1). Thus, FIBCD1 binds to *A. fumigatus* dependent on cell wall PAMP availability.

### FIBCD1 recognizes a composite structure in AIF not exclusively through the S1 binding site

**Figure 3:**
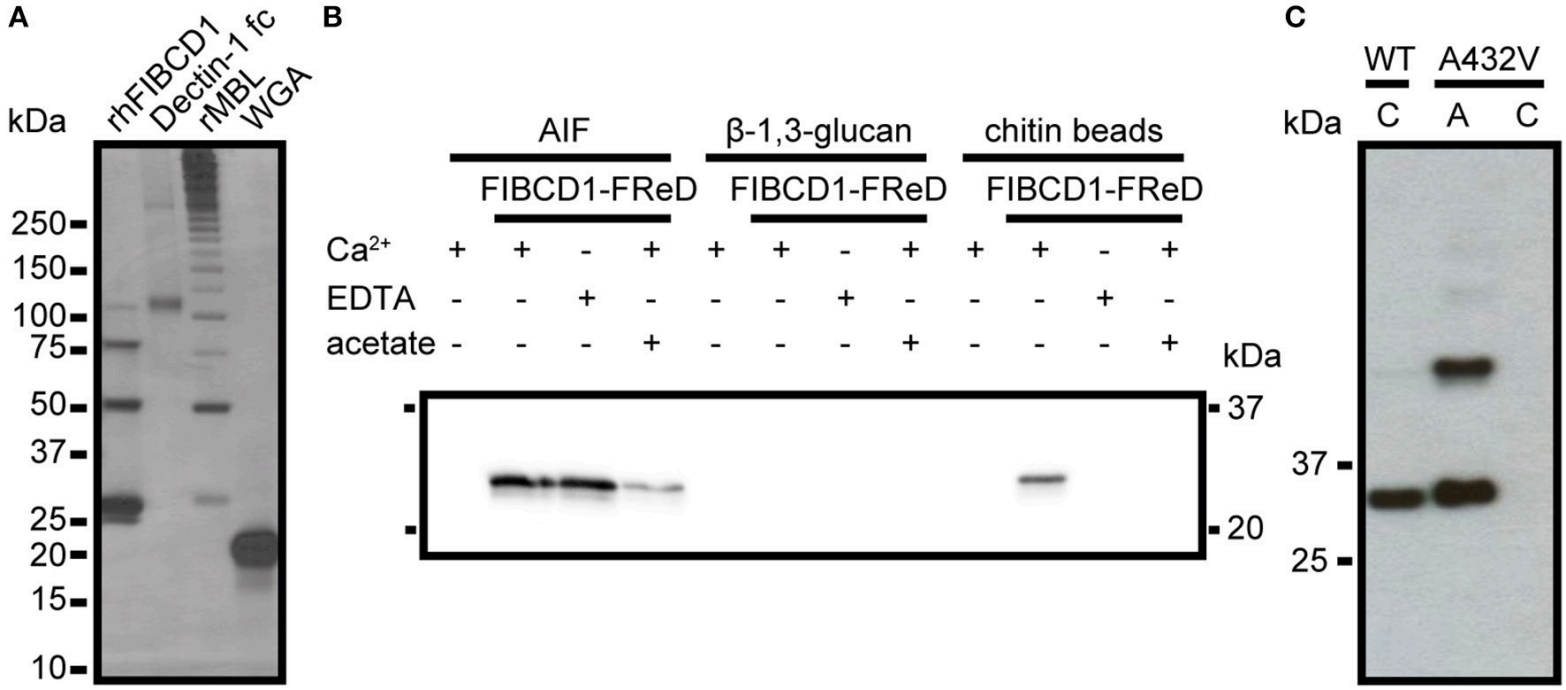
rFIBCD1-FReD binds *A. fumigatus* AIF calcium- and acetate-independently. **(A)** The presence and binding activity of β-1,3-glucan, galactomannan, and chitin in AIF was validated by pull down of know ligands Dectin-1 fc, rMBL, and WGA. The samples were subjected to SDS-PAGE and analyzed by silver staining. Color is adjusted to gray scale. **(B)** Western blot showing FIBCD1-FReD pull down by AIF-associated polysaccharides in the presence of calcium (5 mM CaCl_2_), EDTA (10 mM), and acetate (100 mM). 2 mg of AIF, β-1,3-glucan, and chitin beads were incubated with 5 μg/mL FIBCD1-FReD and pull down was performed as described. Data represent three independent experiments. **(C)** Western blot showing wild type and A432V mutant FIBCD1-FReD pull down by chitin beads and AIF. Color is adjusted to gray scale. Lane 1, 3: chitin beads, Lane 2: AIF.

To evaluate if FIBCD1 binds fungal chitin, we isolated the chitin-containing structural skeleton of the *A. fumigatus* cell wall (AIF) and performed a pull-down assay (Figure 3A). We show that rFIBCD1-FReD binds AIF and pull-down using Dectin-1 fc, rMBL, and WGA demonstrated that cell wall components β-1,3-glucan, galactomannan, and chitin, respectively, were preserved after treatment (12, 26). We observed bands corresponding to FIBCD1-FReD monomeric, dimeric, trimeric, and tetrameric structures, at ~25, 50, 75, and 100 kDa, respectively, and large Dectin-1 fc structures of ~120 and 300 kDa. We observed bands corresponding to higher polymeric structures of rMBL, which assembles into trimers and hexamers of trimers. The smallest rMBL band is ~30 kDa, which is comparable to the rMBL monomer. Finally, we observed a band corresponding to the WGA monomer at ~18 kDa.

FIBCD1 exhibits calcium-dependent binding of chitin and other acetylated compounds through a conserved S1 hydrophobic pocket of the FReD (14, 15). Therefore, we hypothesized that FIBCD1 binds *A. fumigatus* AIF through a similar mechanism. To investigate this, we performed a pull-down assay using AIF in the presence or absence of calcium and acetate (Figure 3B). The insoluble, separate cell wall components β-1,3-glucan and chitin (beads) were used as negative and positive control, respectively (14). Surprisingly, our results show that FIBCD1 binding of AIF is calcium-independent and less sensitive to acetate than purified chitin, suggesting that FIBCD1 binds AIF through additional binding sites outside the S1 binding pocket, potentially involving other polysaccharide structures found in AIF. Therefore, we performed a pull-down assay of wild type FIBCD1-FReD and FIBCD1-FReD with a site-directed mutagenesis abolishing the binding properties of the S1 binding site, A432V (15), using AIF and chitin beads (positive control) (Figure 3C). Our results show that FIBCD1-FReD is capable of binding AIF when the binding activity of the S1 binding site is disrupted.

### FIBCD1 overexpression suppresses IL-8 secretion after stimulation with *A. fumigatus* AIF and galactomannan

**Figure 4:**
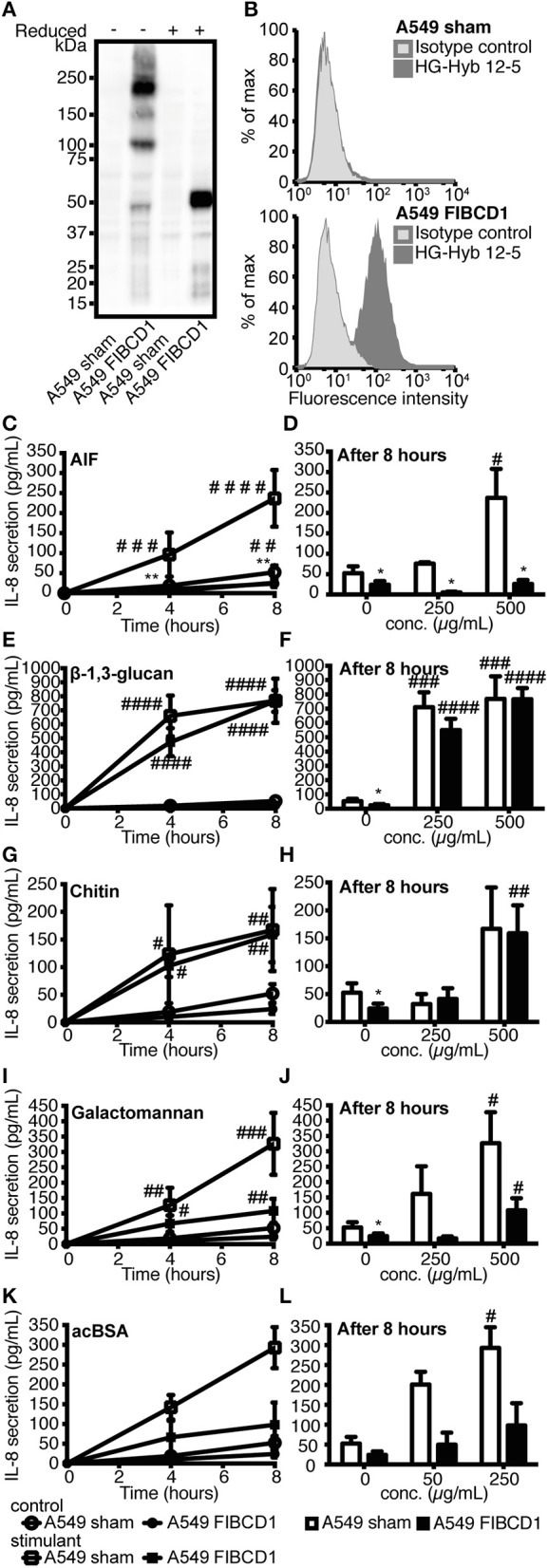
Overexpression of FIBCD1 on the surface of A549 cells inhibits secretion of IL-8. The phenotype of sham- and FIBCD1-transfected A549 cells was analyzed by Western blotting **(A)** and flow cytometry **(B)**. **(A)** Western blot of cell lysate from sham- and FIBCD1-transfected A549 cells under reducing and non-reducing conditions revealed the presence of four known oligomeric forms of FIBCD1 in the FIBCD1-transfected cells when probed with the anti-FIBCD1 mAb clone HG-HYB 12-2. **(B)** Flow cytometry of sham- and FIBCD1-transfected A549 cells showed surface expression of FIBCD1 on FIBCD1-transfected cells and none on sham-transfected cells when using anti-FIBCD1 antibody HG-HYB 12-5 for detection. **(C–L)** A549 sham- and FIBCD1-transfected cells were seeded at a density of one million cells in 2 mL of media per well of a 6-well tissue culture plate and serum-starved overnight prior to stimulation with two different concentrations of several components associated with *A. fumigatus* or FIBCD1 for 0, 4, and 8 h and the concentration of secreted IL-8 was determined by sandwich ELISA as described. Left column: Time-dependent IL-8 secretion to 1,000 μg AIF **(C)**, 1,000 μg β-1,3-glucan **(E)**, 1,000 μg chitin **(G)**, 1,000 μg galactomannan **(I)**, or 500μg acBSA **(K)** per 2 mL medium. Right column: Dose-dependent IL-8 secretion after 8 h of stimulation with AIF **(D)**, β-1,3-glucan **(F)**, chitin **(H)**, galactomannan **(J)**, or acBSA **(L)**. Data are presented as mean ± SEM from three independent experiments. Duplicate cell cultures were used for each of the three independent experiments and ELISA measurements were performed in duplicates on each of these. Data were analyzed by two-way ANOVA, following Tukey's test, ^#^*p* < 0.05, ^##^*p* < 0.01, ^###^*p* < 0.001, and ^####^*p* < 0.0001 relative to 0 h or DPBS-treated cells. *p < 0.05 and **p < 0.01, relative to A549 sham cells stimulated with the same stimulant for the same time period.

A549 lung epithelial cells secrete IL-8 after exposure to *A. fumigatus* irradiated conidia and mycelium (27), and we confirmed a time- and dose-dependent induction of IL-8 following stimulation with live conidia and AIF (Figure S2). Since A549 cells constitutively expressed low or non-detectable levels of FIBCD1 protein (Figure 4B, top panel), we increased FIBCD1 expression by transfecting A549 cells with the full-length human *fibcd1* gene and compared these to sham-transfected A549 cells. In A549 FIBCD1-transfected cells, FIBCD1 protein expression was confirmed by Western blot analysis (Figure 4A) and surface expression by flow cytometry (Figure 4B, bottom panel). Stimulation of the transfected A549 cells with AIF, β-1,3-glucan, chitin, or galactomannan (Figures 4C–L) resulted in time- and dose-dependent IL-8 secretion. FIBCD1 over-expression mediated basal suppression of IL-8 secretion in cells without stimulus (DPBS alone). This FIBCD1-mediated IL-8 suppression was abolished during stimulation with β-1,3-glucan (Figures 4E,F) and chitin (Figures 4G,H). Chitin induced a very low IL-8 response, while β-1,3-glucan induced a high IL-8 response in both cell types. However, FIBCD1-mediated suppression of IL-8 secretion was increased by stimulation with AIF (Figures 4C,D) and galactomannan (Figures 4I,J). A similar response was observed against the positive control ligand acBSA (Figures 4K,L). Hence, FIBCD1 overexpression suppresses IL-8 secretion and maintains this suppression after stimulation with *A. fumigatus* AIF and galactomannan.

### FIBCD1 suppresses mucin and inflammatory gene expression and increases expression of genes involved in mucosal barrier function

**Figure 5:**
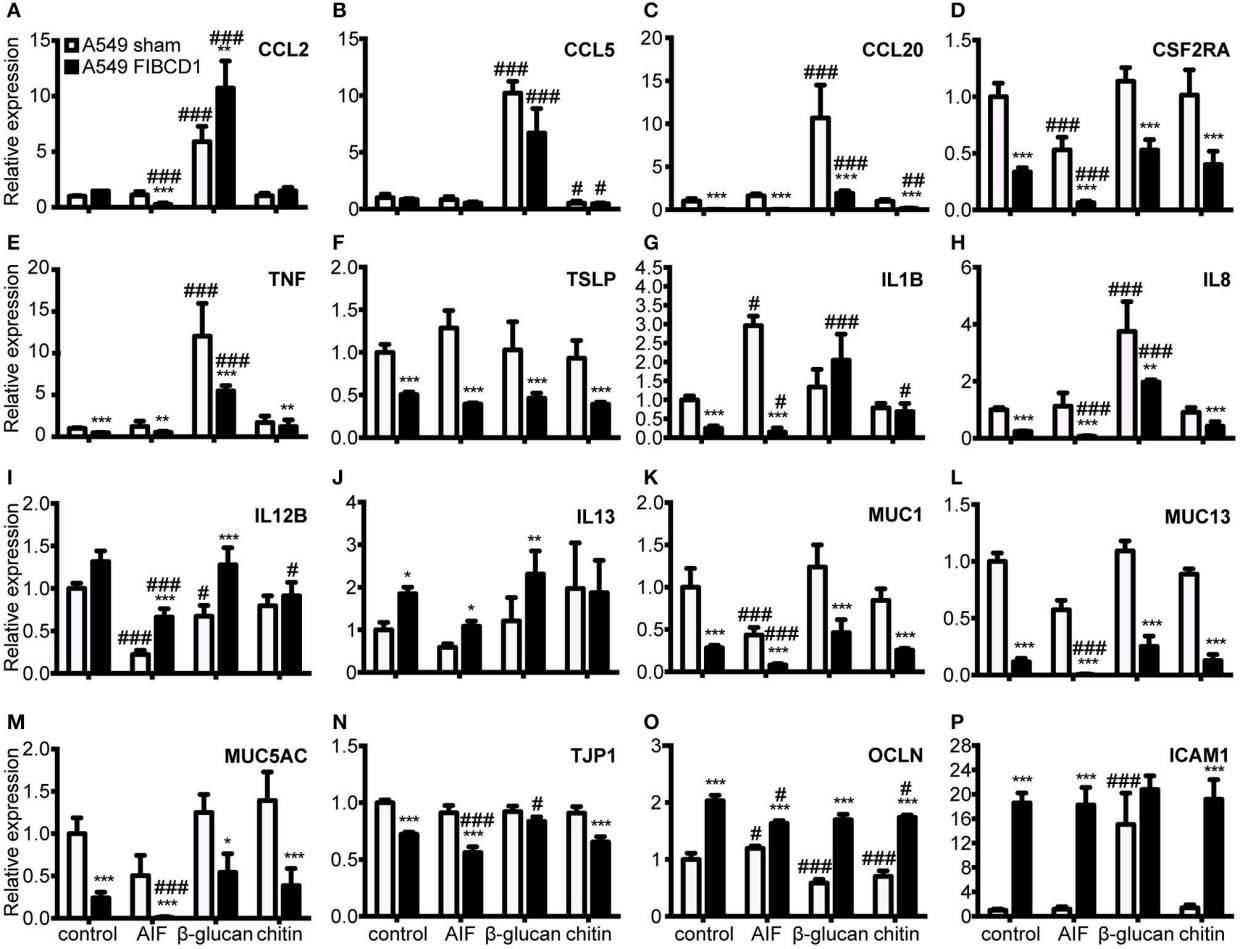
Overexpression of FIBCD1 affects RNA expression of genes associated with immunological responses at mucosal sites. Relative RNA expression of cytokines **(A-J)**, mucins **(K-M)**, TJ proteins **(N-O)**, and adhesion protein **(P)** by A549 sham and A549 FIBCD1 cells in response to stimulation. One million A549 lung epithelial cells transfected with sham and hFIBCD1, respectively, were incubated with DPBS, 500 μg/mL AIF, β-1,3-glucan, and chitin in 2 mL serum-free complete medium for 8 h. The culture supernatants were removed, 1 mL TRIzol added to each well, RNA isolated, cDNA synthetized and tested, and qPCR performed. Data are presented as mean ± SEM from three independent experiments. *P*-values are extracted from the multilevel linear regression models. ^#^*p* < 0.05, ^##^*p* < 0.01, and ^###^*p* < 0.001 relative to DPBS-treated cells. *p < 0.05, **p < 0.01, and ***p < 0.001 relative to A549 sham cells stimulated with the same stimulant.

**Figure 6:**
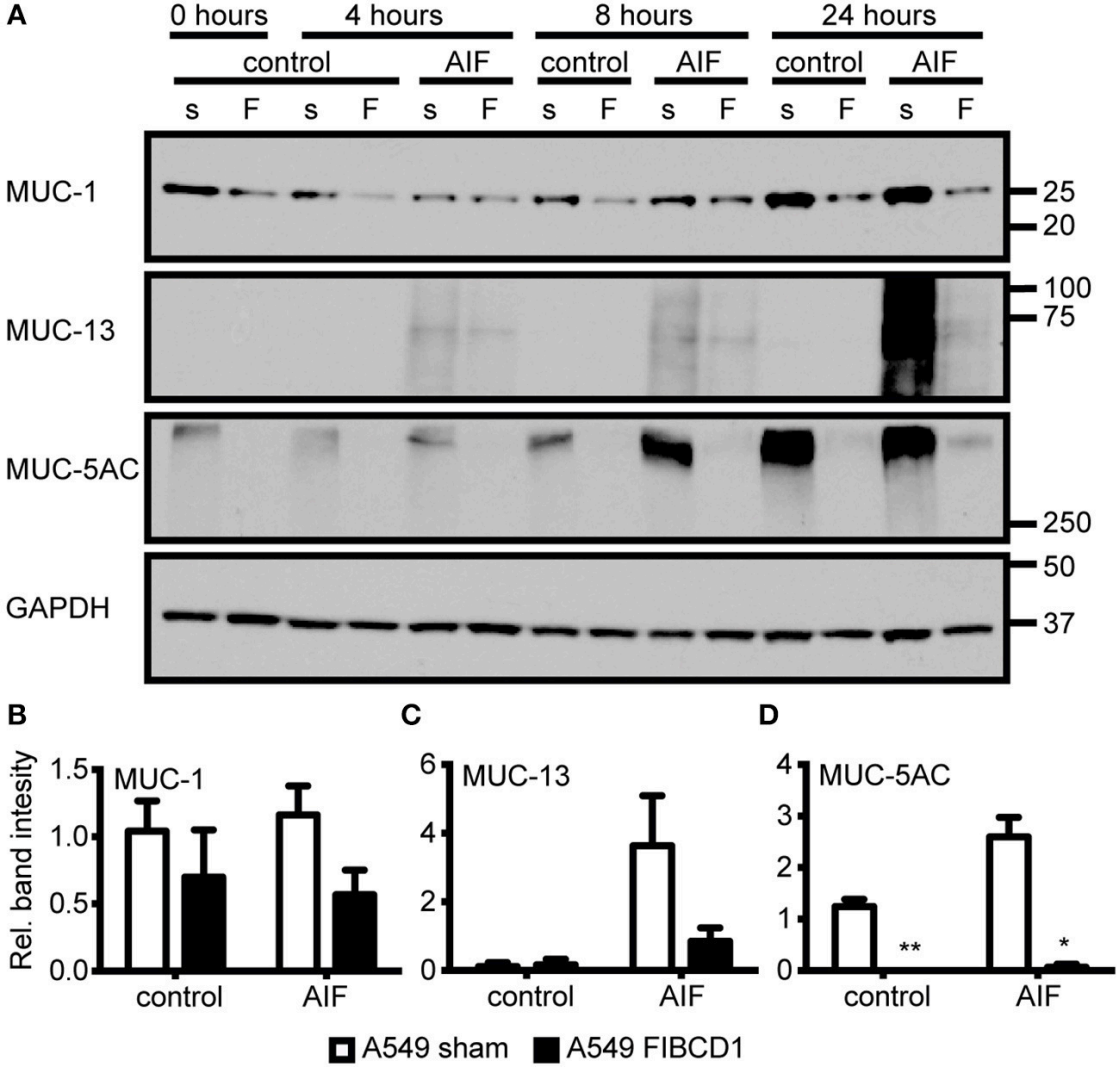
Overexpression of FIBCD1 modulates mucin protein production. A549 sham- and FIBCD1-transfected cells were seeded at a density of one million cells in 2 mL of media per well of a 6-well tissue culture plate and serum-starved over night prior to incubation with DPBS or 500 μg/mL AIF in 2 mL serum-free media for 0, 4, 8, and 24 h. **(A)** Western blot analysis was performed as described using 19 μg of cell lysate per well. Lane 1, 3, 5, 7, 9, 11, 13: A549 sham-transfected cells, Lane 2, 4, 6, 8, 10, 12, 14: A549 FIBCD1-transfected cells. The Western blot shown is from one of three independent experiments yielding similar results. Color is adjusted to gray scale. **(B–D)** Western blots were quantified using ImageJ software. The values obtained after 8 h are expressed as mean ± SEM of the ratio of MUC-1 **(B)**, MUC-13 **(C)**, and MUC-5AC **(D)** to GAPDH from three independent experiments. Data were analyzed by two-way ANOVA, following Tukey's test, ^#^*p* < 0.05, ^##^*p* < 0.01, and ^###^*p* < 0.001 relative to control cells. *p < 0.05, **p < 0.01, and ***p < 0.001 relative to A549 sham cells stimulated with the same stimulant.

Next, we investigated the effect of FIBCD1 transfection on lung epithelial and immune response gene expression after incubation with AIF, β-1,3-glucan, and chitin (Figure 5 and Table S1). Transfected FIBCD1 expression suppressed the RNA expression of proinflammatory cytokines, chemokines, and mucins (CCL20, CSF2RA, TNF, IL1B, IL8, MUC1, MUC13, and MUC5AC) and increased RNA expression of barrier function proteins (OCLN and ICAM1). FIBCD1-mediated suppression of CCL20, IL1B, IL8, and ICAM1 was reversed in the presence of β-1,3-glucan or chitin. In contrast, stimulation with AIF generally increased FIBCD1-mediated suppression of CCL2, CSF2RA, IL1B, IL8, MUC13, and MUC5AC and FIBCD1-mediated induction of the Th2-associated cytokine IL12B. CXCL2, IL6, IL10, IL25, and IL33 were not detected (data not shown). We further evaluated the protein expression of genes with threshold cycle values below 25 (data not shown) regulated at least 2-fold by FIBCD1 expression and not already examined (MUC-1, MUC-13, and MUC-5AC) (Figure 6). In keep with the qPCR data, we observed FIBCD1-mediated suppression of MUC-1,−13, and −5AC in the presence or absence of AIF. Increased FIBCD1-mediated suppression in the presence of AIF was only observed on MUC-13 and −5AC. Thus, FIBCD1 overexpression suppresses mucins and inflammatory gene expression and increases expression of genes involved in mucosal barrier function.

### FIBCD1 influences IL-8 secretion from A549 lung epithelial cells in response to TLR2, TLR4, and TLR5 agonists

**Figure 7:**
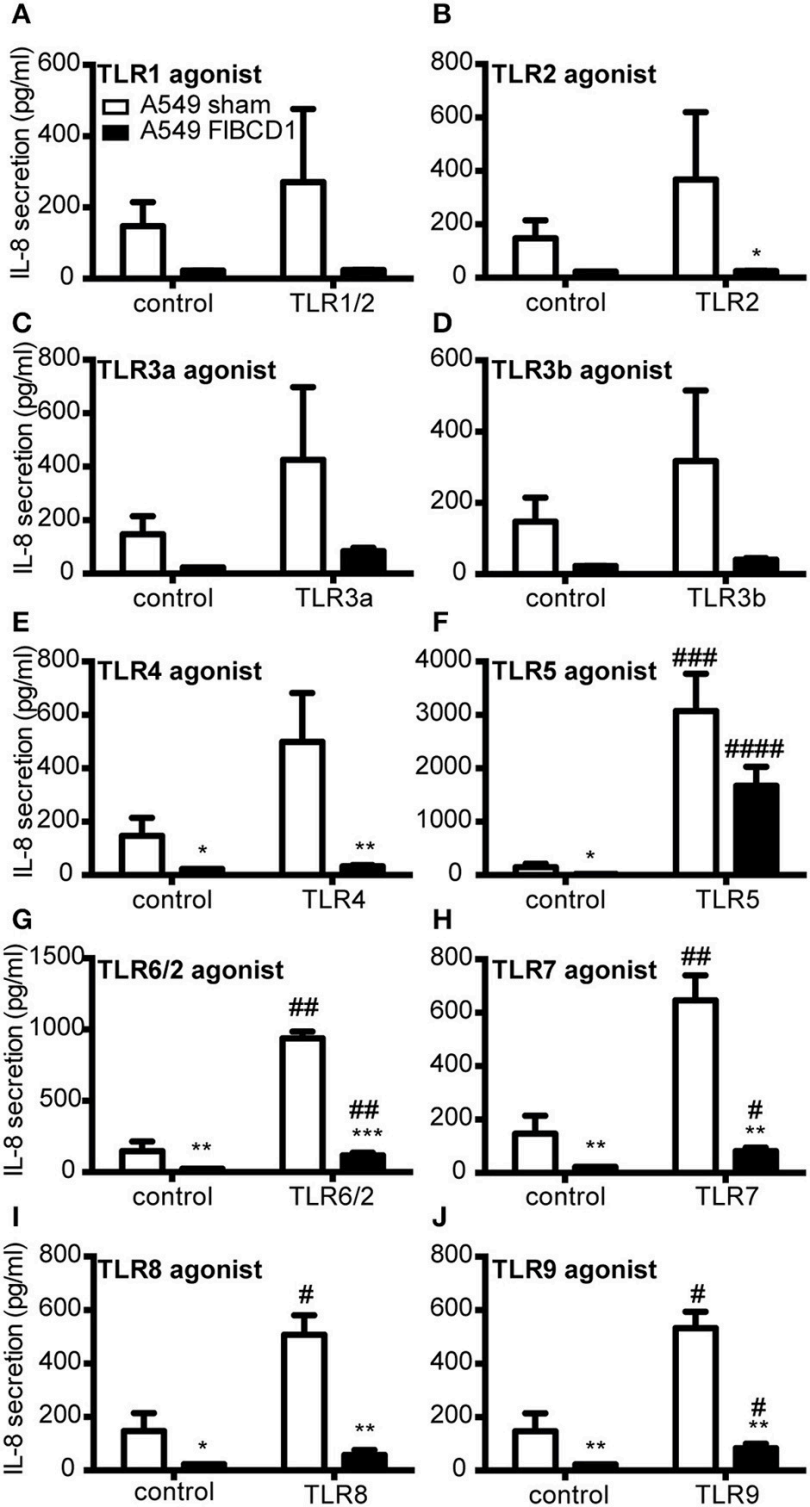
Overexpression of FIBCD1 on the surface of A549 cells inhibits TLR agonist effects. A549 sham- and FIBCD1-transfected cells were seeded at a density of 250,000 cells in 0.5 mL of media per well of a 24-well tissue culture plate and serum-starved overnight prior to stimulation. The cells were stimulated with TLR1/2 **(A)**, 5 **(F)**, and 6/2 **(G)** agonists (0.67 μg/mL), TLR2 **(B)** agonist (6.7·107 cells/mL), TLR3a **(C)** and 3b **(D)** agonists (8.9 μg/mL), TLR4 **(E)** agonist (4.4 μg/mL), TLR7 **(H)** and 8 **(I)** agonists (1.8 μg/mL), and TLR9 **(J)** agonist (0.068 μg/mL) for 8 h and the concentration of secreted IL-8 was determined by sandwich ELISA as described in methods. Data are presented as mean ± SEM from three independent experiments. Duplicate cell cultures were used for each of the three independent experiments and ELISA measurements were performed in duplicates on each of these. Data were analyzed by two-way ANOVA, followed by Tukey's post-test, ^#^*p* < 0.05, ^##^*p* < 0.01, ^###^*p* < 0.001, and ^####^*p* < 0.0001 relative to DPBS-treated cells. *p < 0.05, **p < 0.01, and ***p < 0.001 relative to A549 sham cells stimulated with the same stimulant.

Finally, we examined whether FIBCD1 expression modulated TLR-induced IL-8 secretion by stimulating A549 sham- and FIBCD1-transfected cells with 10 different TLR agonists and measuring IL-8 levels in culture supernatants (Figure 7). We found that FIBCD1 in particular suppressed TLR2 and −4 agonist-induced IL-8 secretion and that TLR5 agonist circumvented IL-8 suppression. These findings are supported by qPCR (Figure S4). Though TLR6/2,−7,−8, and −9 agonist-induced IL-8 secretion was suppressed by FIBCD1 expression, it differed little from the basal suppression. Contrary to this, TLR2,−4, and −5 agonist-induced IL-8 secretion was changed at least 2-fold from basal suppression. Similar results were observed after 4 h of stimulation (Figure S3). Hence, FIBCD1 expression influences TLR2,−4, and −5 agonist-induced IL-8 secretion.

## Discussion

We previously reported FIBCD1 protein expression at the brush border of human intestinal epithelium and identified binding of crab shell chitin in the S1 binding site of FIBCD1-FReD (14, 15). In the current study, we observed FIBCD1 protein expression at the apical site of human bronchial and alveolar epithelium and binding between FIBCD1 and *A. fumigatus* dependent on cell wall availability, as well as an S1 site-independent AIF recognition. We observed FIBCD1-mediated suppression of IL-8 secretion, mucin production, and transcription of genes involved in inflammatory signaling and FIBCD1-mediated induction of tight junction and cell adhesion molecule transcription. Additionally, we show that this regulation was modulated by stimulation with *A. fumigatus* cell wall and individual cell wall components, i.e., β-1,3-glucan and chitin, and FIBCD1 expression effected TLR agonist-induced IL-8 secretion.

Fluorescence microscopy of different *A. fumigatus* morphotypes (Figure 2) showed that soluble FIBCD1 binding was dependent on conidial germination and concomitant exposure of cell wall polysaccharides. Thus, FIBCD1's role in the immune response against *A. fumigatus* may be associated with the expanded immune response, i.e. opsonization and chemotactic attraction of neutrophils to the site of inflammation (1). Furthermore, we observed co-localization between chitin-rich zones and areas recognized by FIBCD1, which led us to investigate interaction between FIBCD1 and the chitin-containing structural skeleton of the *A. fumigatus* cell wall (AIF). Pull-down experiments (Figure 3) revealed that FIBCD1 recognizes AIF independently of calcium and the S1 binding site with less sensitivity to acetate compared to that of chitin.

Chitin is the only component of the cell wall known as a FIBCD1 ligand, which it binds through the S1 binding site (14). These findings support that FIBCD1 recognizes fungal chitin through the S1 binding site, but also recognizes other cell wall structures found in AIF, possibly through conjunctional binding sites or separately in other unknown binding sites.

Similar to a previous study using A549 lung epithelial cells (27), we observed a time- and dose-dependent secretion of IL-8, a proinflammatory chemokine that attracts neutrophils and thereby plays an important role in the inflammatory response against *A. fumigatus* hyphae (1). Stimulation of A549 lung epithelial cells overexpressing FIBCD1 (Figure 4) revealed a suppression of IL-8 secretion, which is abolished by stimulation with β-1,3-glucan and chitin and increased by AIF, galactomannan, and acBSA. As a non-FIBCD1 binding ligand, we expect the observed effect of β-1,3-glucan on FIBCD1-mediated suppression of IL-8 secretion to be caused by the activation of pathways that either deactivate FIBCD1, decrease its expression, or induce IL-8 production through circumventing pathways, possibly through the well-known β-1,3-glucan PRR Dectin-1 (12). Different effects of the FIBCD1 ligands AIF, chitin, and acBSA were observed, which is likely caused by particle size and source differences known to impact epithelial responses against chitin (28, 29). Previous studies have shown that large particles fail to induce an inflammatory response, while intermediate and small particles protect against Th2-associated allergy (28, 29). In the current study, the exact size of the chitin particles is unknown, however, large, insoluble fragments were observed by bright field microscopy during the stimulation study, which may have effected the interaction between FIBCD1 and chitin. Contrary to this, AIF was administered in intermediate particle size (<40 μm) and acBSA and galactomannan are both soluble molecules. It is currently unclear if the FIBCD1-mediated suppression of IL-8 secretion in response to galactomannan is caused by mere particle size, intersections of signaling pathways or by direct or indirect interaction between FIBCD1 and galactomannan. It is, however, clear that galactomannan does not bind to the S1 binding site of FIBCD1-FReD (Figure S5).

We observed a FIBCD1-mediated transcriptional suppression of proinflammatory cytokines, chemokines, and mucins and an induction of barrier function gene expression (Figure 5). During the anti-fungal response, several proteins of the genes suppressed by FIBCD1 are released from epithelium to aid in the response. They cause increased inflammation, recruitment of neutrophils, monocytes, and mast cells to the site of inflammation, stimulation of naïve Th cell polarization to Th2 cells, and facilitate homing of these cells. Finally, they induce secretion of MUC-5AC and epithelial expression of MUC-1 and −13, and disrupt epithelial apical junctional complexes, which further enhances inflammation and antigen presentation by dendritic cells (30–40). Thus, FIBCD1 may serve as a general suppressor of airway inflammation. Similar to the IL-8 secretion observations, stimulation with β-1,3-glucan and chitin diminish and AIF increases FIBCD1-mediated suppression.

Mucociliary clearance is the primary defense mechanism against *A. fumigatus* in the bronchioles (6) and is highly dependent on the synthesis, glycosylation, and release of secreted and membrane-bound mucins (37, 41). FIBCD1-mediated suppression of MUC-1 and MUC-5AC and an AIF-increased FIBCD1-mediated suppression of MUC-13 and MUC-5AC were observed by Western blotting of cell lysates from A549 lung epithelial cells (Figure 6). This indicates that FIBCD1 expression impacts mucus composition and thereby fungal clearance. However, whether these alterations are beneficial for fungi or host is unclear. Additionally, MUC-5AC is a secretory mucin (41) and the lack of vesicle storage in the A549 FIBCD1-transfected cells compared to the A549 sham-transfected cells may reflect increased release as well as decreased production.

Finally, we observed that FIBCD1 overexpression affects the inflammatory response mediated by TLRs in A549 lung epithelial cells (Figure 7). The overexpression decreases TLR2- and TLR4-induced IL-8 secretion and increases TLR5-induced IL-8 secretion. Intriguingly, these TLRs are associated with different outcomes of fungal infections. Several studies have investigated the role of TLR2 and TLR4 in the immunological response against *A. fumigatus* and found that their expression is required for optimal host defense (6, 42, 43). The investigation of TLR5's involvement in anti-*A. fumigatus* responses is fairly limited, and it has early been demonstrated that TLR5 have no influence on the anti-*A. fumigatus* response (42). However, Rodland et al. (44) observed an increased RNA expression of the receptor in human monocytes challenges with *A. fumigatus* and later found that TLR5 has an enhancing effect on the viability of conidia (45).

Collectively, our findings demonstrate FIBCD1 as human lung epithelial PRR that recognizes the cell wall of *A. fumigatus* and suppresses epithelial inflammatory signaling and mucin production. FIBCD1 activation may have a beneficial effect by decreasing inflammatory damage or an adverse effect by diminishing anti-fungal responses such as recruitment of neutrophils and mucin production.

## Author contributions

The experiments were designed by CJ, LD, AS, GS, ST, and UH. CJ, LD, KC, JM, MH, and ON performed the experiments. CJ performed statistical analysis of the data. The reagents, materials, and analysis tools were provided by ON, AS, GS, and UH. CJ, ST, and UH wrote the paper.

### Conflict of interest statement

The authors declare that the research was conducted in the absence of any commercial or financial relationships that could be construed as a potential conflict of interest. The handling editor is currently co-organizing a Research Topic with one of the authors ST, and confirms the absence of any other collaboration.

## Supplementary material

Figure S1 FIBCD1 does not recognizes *A. fumigatus* resting conidia. Bright field **(A)** and fluorescence **(B)** microscopy of *A. fumigatus*. **(A,B)** *A. fumigatus* conidia/mL was grown in SD medium to form fungal hyphae followed by staining with Alexa 488-labeled FIBCD1 as described in materials and methods. A clear recognition of fungal hyphae by FIBCD1 was observed while no staining was seen of the resting conidia indicated by arrows was seen. Image: 20X objective.

Figure S2 *A. fumigatus* conidia and AIF induce IL-8 secretion by A549 wild type cells time- and dose-dependently. Wild type A549 cells were seeded at a density of 3·10^5^ cells in 2 mL of media per well of a 12-well tissue culture plate and serum-starved over night prior to stimulation and the concentration of secreted IL-8 was determined by sandwich ELISA as described in methods. Left panels: Time-dependent IL-8 secretion to 3·10^5^ conidia **(A)** and 800μg AIF **(C)** per 2 mL medium. Right panels: Dose-dependent IL-8 secretion after 8 h of stimulation with conidia **(B)** and AIF **(D)**. Data are presented as mean ± SEM from three independent experiments. ELISA measurements were performed in triplicates **(A,C)** and duplicates **(B,D)** for each of the three independent experiments. Data were analyzed by two-way **(A,C)** and one-way **(B,D)** ANOVA, followed by Tukey's post-test. ^*^*p* < 0.05, ^**^*p* < 0.01, ^***^*p* < 0.001, and ^****^*p* < 0.0001 relative to previous time or dosage.

Figure S3 Overexpression of FIBCD1 on the surface of A549 cells influences TLR agonist effects after 4 h. A549 sham- and FIBCD1-transfected cells were seeded at a density of 250,000 cells in 0.5 mL of media per well of a 24-well tissue culture plate and serum-starved overnight prior to stimulation. The cells were stimulated with TLR1/2 **(A)**, 5 **(F)**, and 6/2 **(G)** agonists (0.67 μg/mL), TLR2 **(B)** agonist (6.7·107 cells/mL), TLR3a **(C)** and 3b **(D)** agonists (8.9 μg/mL), TLR4 **(E)** agonist (4.4 μg/mL), TLR7 **(H)** and 8 **(I)** agonists (1.8 μg/mL), and TLR9 **(J)** agonist (0.068 μg/mL) for 4 h and the concentration of secreted IL-8 was determined by sandwich ELISA as described in methods. Data are presented as mean ± SEM from three independent experiments. Duplicate cell cultures were used for each of the three independent experiments and ELISA measurements were performed in duplicates on each of these. Data were analyzed by two-way ANOVA, following Tukey's test, ^#^*p* < 0.05, ^##^*p* < 0.01, ^###^*p* < 0.001, and ^####^*p* < 0.001 relative to DPBS-treated cells. ^*^*p* < 0.05, ^**^*p* < 0.01, and ^***^*p* < 0.001 relative to A549 sham cells stimulated with the same stimulant.

Figure S4 Overexpression of FIBCD1 on the surface of A549 cells influences TLR agonist effects. A549 sham- and FIBCD1-transfected cells were seeded at a density of 250,000 cells in 0.5 mL of media per well of a 24-well tissue culture plate and serum-starved overnight prior to stimulation. The cells were stimulated with TLR1/2 and 5 agonists (0.67 μg/mL), TLR2 agonist (6.7·10^7^ cells/mL), and TLR4 agonist (4.4 μg/mL) for 4 h **(A)** and 8 h **(B)**. The culture supernatants were removed, 0.5 mL TRIzol added to each well, RNA isolated, cDNA synthetized, and qPCR performed. Data are presented as mean ± SEM from three independent experiments and qPCR measurements were performed in duplicates on each of these. Data were analyzed by two-way ANOVA, following Tukey's test, ^#^*p* < 0.05, ^##^*p* < 0.01 and ^###^*p* < 0.001, relative to DPBS-treated cells. ^**^*p* < 0.01, and relative to A549 sham cells stimulated with the same stimulant.

Figure S5 Competitive ELISA showing galactomannan's effect on binding between acBSA and FIBCD1-FReD. A maxisorp immuno plate was coated with 1 μg/mL acBSA in ELISA coating buffer overnight. PBS, acetate, mannan, and galactomannan were loaded in a 2-fold dilution series in TBS/0.05% tween/5 mM CaCl_2_ starting at 100 mM, 2 mg/mL, and 2 mg/mL, respectively, along with 0.5 μg/mL FIBCD1-FReD. PBS was used as a control for decreased Ca^2+^ presence by the addition of polysaccharides suspended in PBS, calcium content started at 2.5 mM CaCl_2_. FIBCD1-FReD was detected by 1 μg/mL HG-HYB-12-6 in TBS/0.05% tween/5 mM CaCl_2_ and HRP-conjugated rabbit anti-mouse antibody. Data represent three independent experiments and is shown as mean ± SEM. ELISA measurements were performed in duplicates for each of the three independent experiments.

Table S1 Multilevel linear regression models. Results of the multilevel linear regression models used to analyze relative mRNA expression of cytokines, mucins, adhesion proteins, and TJ proteins in A549 sham and A549 FIBCD1 cells in response to stimulation (Figure 6).

## Abbreviations

acBSA: acetylated BSA
*A. fumigatus*: *Aspergillus fumigatus*
AIF: alkali-insoluble fraction
CNRQ: calibrated normalized relative quantity
DPBS: Dulbecco's PBS
FIBCD1: fibrinogen C domain-containing protein 1
FReD: fibrinogen-related domain
IAA: iodoacetamide
IHC: Immunohistochemical
MBL: mannan-binding lectin
MOPS: 3-(N-morpholino)propanesulfonic acid
MUC: mucin
PAMPs: pathogen-associated molecular patterns
PRR: pattern recognition receptor
qPCR: quantitative PCR
RT: room temperature
SD: sabouraud dextrose
WGA: wheat germ agglutinin.
