# Supplementary figures and images for: FIBCD1 Binds Aspergillus fumigatus and Regulates Lung Epithelial Response to Cell Wall Components
Source: Front Immunol. 2018 Sep 18;9:1967. doi: 10.3389/fimmu.2018.01967 (PMC6153955; doi:10.3389/fimmu.2018.01967)

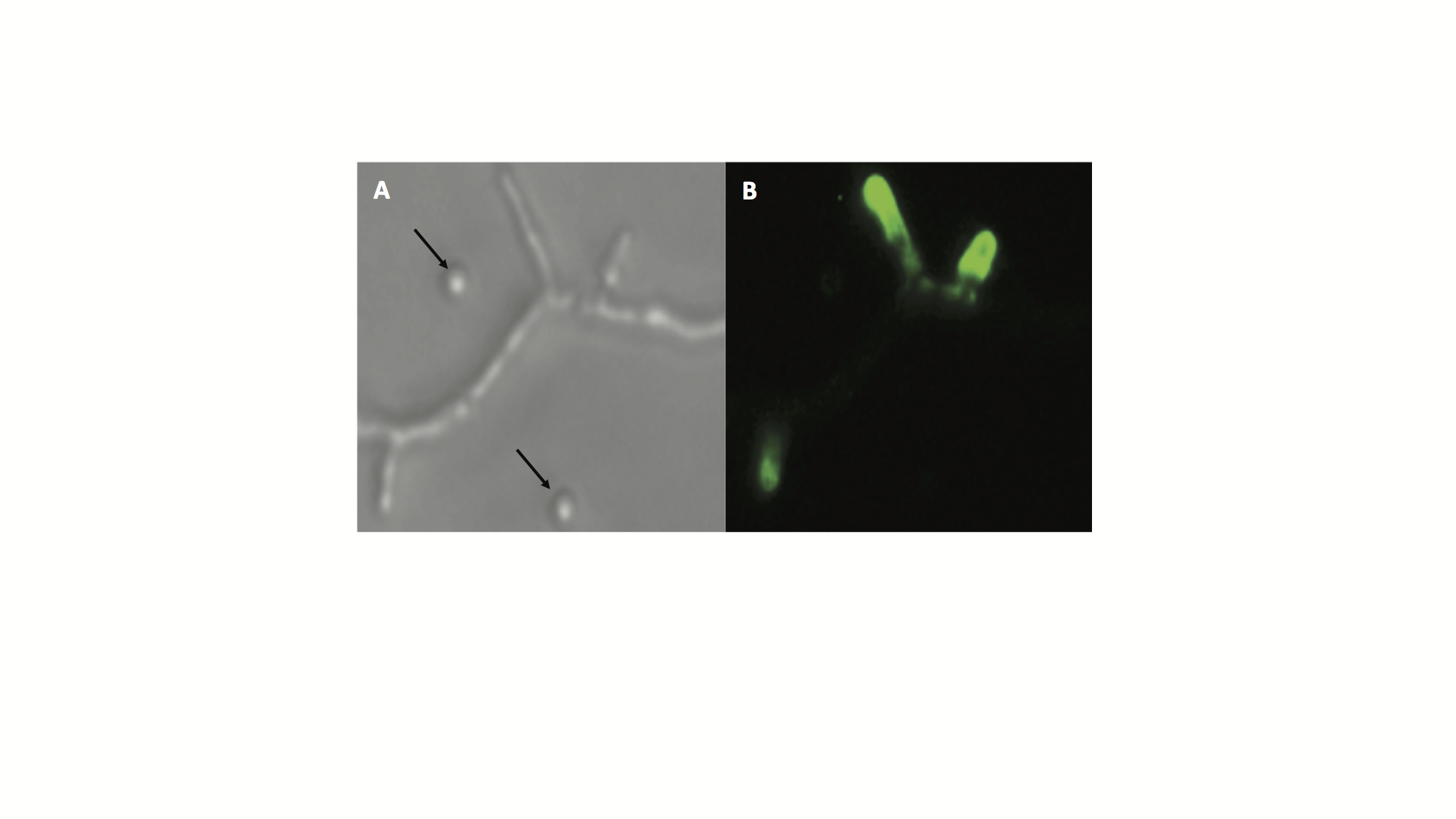

Supplement: Figure S1 — FIBCD1 does not recognizes A. fumigatus resting conidia. Bright field (A) and fluorescence (B) microscopy of A. fumigatus. (A,B) A. fumigatus conidia/mL was grown in SD medium to form fungal hyphae followed by staining with Alexa 488-labeled FIBCD1 as described in materials and methods. A clear recognition of fungal hyphae by FIBCD1 was observed while no staining was seen of the resting conidia indicated by arrows was seen. Image: 20X objective. [file Image_1.TIF]

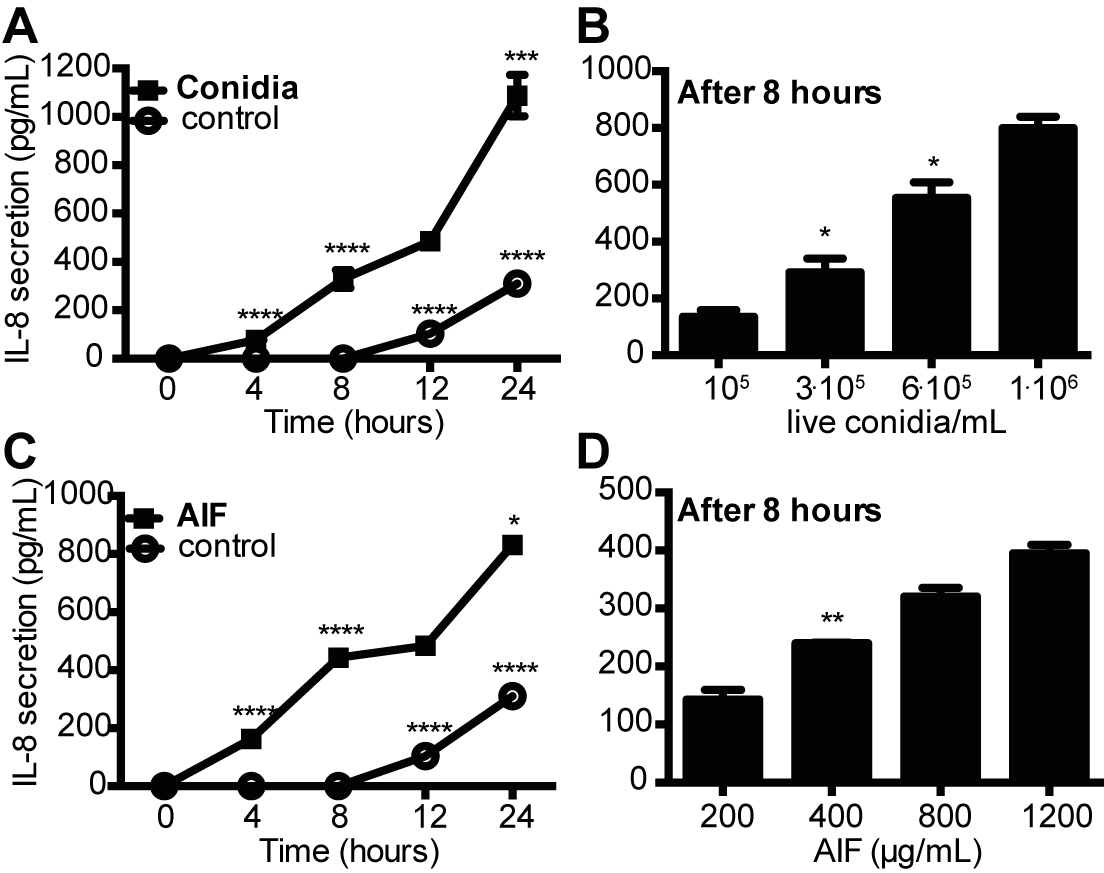

Supplement: Figure S2 — A. fumigatus conidia and AIF induce IL-8 secretion by A549 wild type cells time- and dose-dependently. Wild type A549 cells were seeded at a density of 3·105 cells in 2 mL of media per well of a 12-well tissue culture plate and serum-starved over night prior to stimulation and the concentration of secreted IL-8 was determined by sandwich ELISA as described in methods. Left panels: Time-dependent IL-8 secretion to 3·105 conidia (A) and 800μg AIF (C) per 2 mL medium. Right panels: Dose-dependent IL-8 secretion after 8 h of stimulation with conidia (B) and AIF (D). Data are presented as mean ± SEM from three independent experiments. ELISA measurements were performed in triplicates (A,C) and duplicates (B,D) for each of the three independent experiments. Data were analyzed by two-way (A,C) and one-way (B,D) ANOVA, followed by Tukey's post-test. *p < 0.05, **p < 0.01, ***p < 0.001, and ****p < 0.0001 relative to previous time or dosage. [file Image_2.TIF]

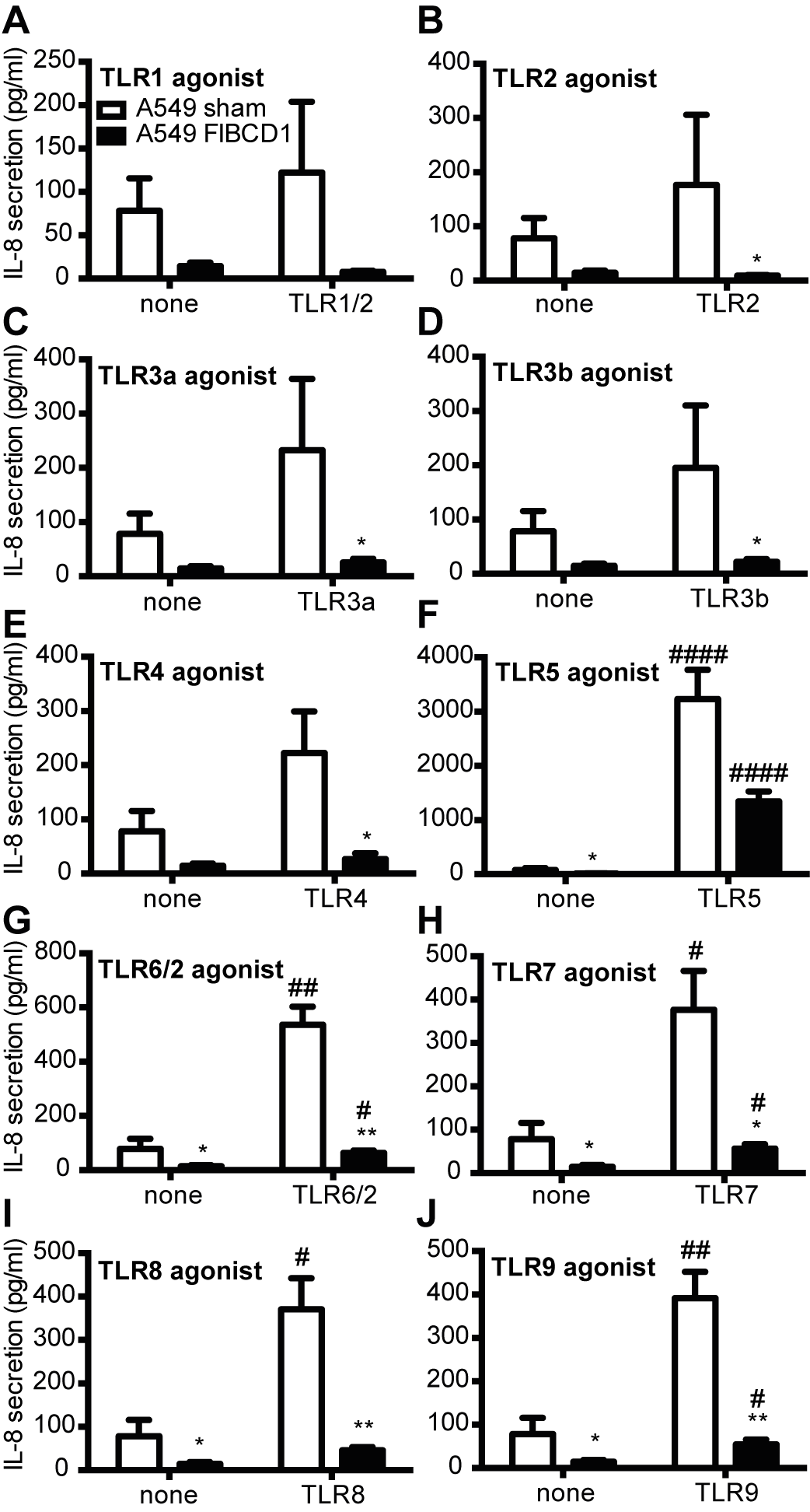

Supplement: Figure S3 — Overexpression of FIBCD1 on the surface of A549 cells influences TLR agonist effects after 4 h. A549 sham- and FIBCD1-transfected cells were seeded at a density of 250,000 cells in 0.5 mL of media per well of a 24-well tissue culture plate and serum-starved overnight prior to stimulation. The cells were stimulated with TLR1/2 (A), 5 (F), and 6/2 (G) agonists (0.67 μg/mL), TLR2 (B) agonist (6.7·107 cells/mL), TLR3a (C) and 3b (D) agonists (8.9 μg/mL), TLR4 (E) agonist (4.4 μg/mL), TLR7 (H) and 8 (I) agonists (1.8 μg/mL), and TLR9 (J) agonist (0.068 μg/mL) for 4 h and the concentration of secreted IL-8 was determined by sandwich ELISA as described in methods. Data are presented as mean ± SEM from three independent experiments. Duplicate cell cultures were used for each of the three independent experiments and ELISA measurements were performed in duplicates on each of these. Data were analyzed by two-way ANOVA, following Tukey's test, #p < 0.05, ##p < 0.01, ###p < 0.001, and ####p < 0.001 relative to DPBS-treated cells. *p < 0.05, **p < 0.01, and ***p < 0.001 relative to A549 sham cells stimulated with the same stimulant. [file Image_3.TIF]

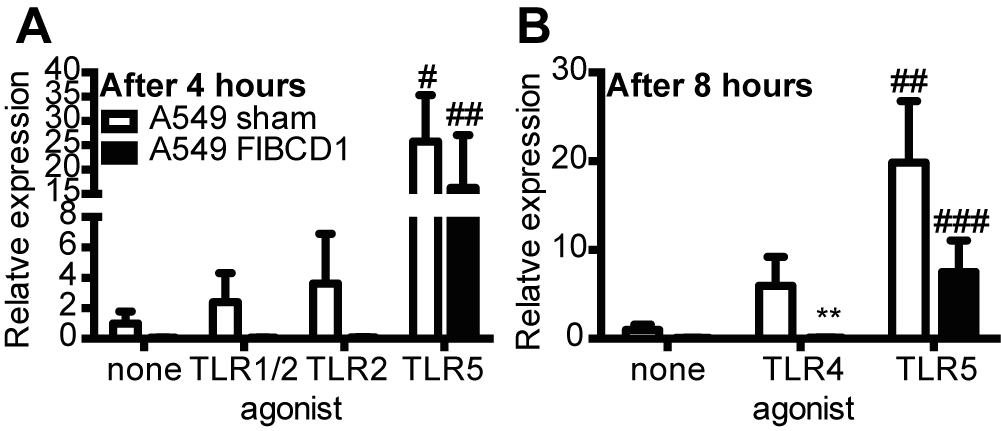

Supplement: Figure S4 — Overexpression of FIBCD1 on the surface of A549 cells influences TLR agonist effects. A549 sham- and FIBCD1-transfected cells were seeded at a density of 250,000 cells in 0.5 mL of media per well of a 24-well tissue culture plate and serum-starved overnight prior to stimulation. The cells were stimulated with TLR1/2 and 5 agonists (0.67 μg/mL), TLR2 agonist (6.7·107 cells/mL), and TLR4 agonist (4.4 μg/mL) for 4 h (A) and 8 h (B). The culture supernatants were removed, 0.5 mL TRIzol added to each well, RNA isolated, cDNA synthetized, and qPCR performed. Data are presented as mean ± SEM from three independent experiments and qPCR measurements were performed in duplicates on each of these. Data were analyzed by two-way ANOVA, following Tukey's test, #p < 0.05, ##p < 0.01 and ###p < 0.001, relative to DPBS-treated cells. **p < 0.01, and relative to A549 sham cells stimulated with the same stimulant. [file Image_4.TIF]

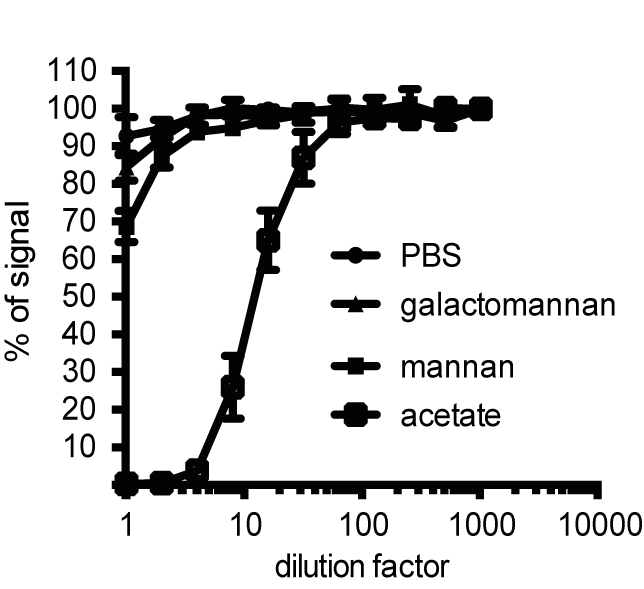

Supplement: Figure S5 — Competitive ELISA showing galactomannan's effect on binding between acBSA and FIBCD1-FReD. A maxisorp immuno plate was coated with 1 μg/mL acBSA in ELISA coating buffer overnight. PBS, acetate, mannan, and galactomannan were loaded in a 2-fold dilution series in TBS/0.05% tween/5 mM CaCl2 starting at 100 mM, 2 mg/mL, and 2 mg/mL, respectively, along with 0.5 μg/mL FIBCD1-FReD. PBS was used as a control for decreased Ca2+ presence by the addition of polysaccharides suspended in PBS, calcium content started at 2.5 mM CaCl2. FIBCD1-FReD was detected by 1 μg/mL HG-HYB-12-6 in TBS/0.05% tween/5 mM CaCl2 and HRP-conjugated rabbit anti-mouse antibody. Data represent three independent experiments and is shown as mean ± SEM. ELISA measurements were performed in duplicates for each of the three independent experiments. [file Image_5.TIF]
