# Supplementary material for: FIBCD1 Binds Aspergillus fumigatus and Regulates Lung Epithelial Response to Cell Wall Components
Source: Front Immunol. 2018 Sep 18;9:1967. doi: 10.3389/fimmu.2018.01967 (PMC6153955; doi:10.3389/fimmu.2018.01967)
Supplement: Table S1 — Multilevel linear regression models. Results of the multilevel linear regression models used to analyze relative mRNA expression of cytokines, mucins, adhesion proteins, and TJ proteins in A549 sham and A549 FIBCD1 cells in response to stimulation (Figure 6). [file Data_Sheet_1.PDF]

Variable Log2(CNRQ)		mean	SD	p	95% CI
**CCL2**					
Ligand					
	AIF	0.049	0.452	0.914	-0.837 ; 0.934
	curdlan	2.597	0.436	0.000	1.742 ; 3.452
	chitin	-0.175	0.436	0.688	-1.031 ; 0.680
Genotype					
	FIBCD1	0.554	0.285	0.052	-0.005 ; 1.113
Interaction					
	AIF#FIBCD1	-2.910	0.403	0.000	-3.700 ; -2.120
	curdlan#FIBCD1	0.331	0.403	0.412	-0.460 ; 1.121
	chitin#FIBCD1	-0.054	0.403	0.893	-0.844 ; 0.736
_cons		-0.003	0.319	0.992	-0.629 ; 0.623
**CCL5**					
Ligand					
	AIF	-0.365	0.464	0.431	-1.275 ; 0.544
	curdlan	3.517	0.459	0.000	2.616 ; 4.417
	chitin	-1.089	0.459	0.018	-1.989 ; -0.188
Genotype					
	FIBCD1	-0.130	0.382	0.735	-0.879 ; 0.619
Interaction					
	AIF#FIBCD1	-0.401	0.540	0.458	-1.460 ; 0.658
	curdlan#FIBCD1	-0.609	0.540	0.260	-1.668 ; 0.450
	chitin#FIBCD1	0.073	0.540	0.892	-0.986 ; 1.132
_cons		-0.140	0.328	0.671	-0.783 ; 0.503
**CCL20**					
Ligand					
	AIF	0.843	0.485	0.082	-0.108 ; 1.794
	curdlan	3.314	0.485	0.000	2.363 ; 4.264
	chitin	0.025	0.485	0.959	-0.926 ; 0.976
Genotype					
	FIBCD1	-4.583	0.469	0.000	-5.501 ; -3.665
Interaction					
	AIF#FIBCD1	-0.858	0.663	0.196	-2.156 ; 0.441
	curdlan#FIBCD1	2.329	0.663	0.000	1.030 ; 3.628
	chitin#FIBCD1	1.656	0.663	0.012	0.357 ; 2.954
_cons		-0.161	0.343	0.640	-0.833 ; 0.512
**CSF2RA**					
Ligand					
	AIF	-0.952	0.352	0.007	-1.643 ; -0.262
	curdlan	0.103	0.328	0.753	-0.539 ; 0.746
	chitin	0.153	0.328	0.641	-0.490 ; 0.796
Genotype					

	FIBCD1	-1.574	0.143	0.000	-1.853	;	-1.294
Interaction							
	AIF#FIBCD1	-1.486	0.202	0.000	-1.882	;	-1.091
	curdlan#FIBCD1	0.428	0.202	0.034	0.033	;	0.823
	chitin#FIBCD1	0.159	0.202	0.430	-0.236	;	0.554
_cons		-0.023	0.249	0.928	-0.511	;	0.466
**TNF**							
Ligand							
	AIF	-0.057	0.686	0.934	-1.401	;	1.288
	curdlan	3.950	0.643	0.000	2.689	;	5.212
	chitin	0.011	0.643	0.986	-1.250	;	1.272
Genotype							
	FIBCD1	-1.053	0.310	0.001	-1.661	;	-0.445
Interaction							
	AIF#FIBCD1	0.082	0.439	0.852	-0.778	;	0.941
	curdlan#FIBCD1	0.051	0.439	0.908	-0.809	;	0.910
	chitin#FIBCD1	0.225	0.439	0.609	-0.635	;	1.084
_cons		-0.010	0.485	0.984	-0.960	;	0.941
**TSLP**							
Ligand							
	AIF	0.341	0.279	0.221	-0.205	;	0.888
	curdlan	-0.114	0.276	0.679	-0.655	;	0.427
	chitin	-0.244	0.276	0.378	-0.784	;	0.297
Genotype							
	FIBCD1	-0.974	0.227	0.000	-1.420	;	-0.528
Interaction							
	AIF#FIBCD1	-0.707	0.322	0.028	-1.734	;	-0.076
	curdlan#FIBCD1	-0.046	0.322	0.885	-0.677	;	0.584
	chitin#FIBCD1	-0.208	0.322	0.517	-0.839	;	0.422
_cons		-0.013	0.197	0.947	-0.400	;	0.374
**IL1B**							
Ligand							
	AIF	1.573	0.674	0.020	0.253	;	2.894
	curdlan	0.224	0.668	0.737	-1.085	;	1.534
	chitin	-0.397	0.668	0.552	-1.706	;	0.912
Genotype							
	FIBCD1	-2.088	0.566	0.000	-3.196	;	-0.979
Interaction							
	AIF#FIBCD1	-3.165	0.800	0.000	-4.732	;	-1.597
	curdlan#FIBCD1	2.669	0.800	0.001	1.102	;	4.237
	chitin#FIBCD1	1.736	0.800	0.030	0.168	;	3.303
_cons		-0.016	0.476	0.974	-0.949	;	0.918
**IL8**							
Ligand							

	AIF	-0.049	0.470	0.916	-0.970 ;	0.871
	curdlan	1.867	0.451	0.000	0.983 ;	2.751
	chitin	-0.303	0.451	0.502	-1.187 ;	0.582
Genotype						
	FIBCD1	-2.075	0.281	0.000	-2.625 ;	-1.525
Interaction						
	AIF#FIBCD1	-1.972	0.397	0.000	-2.749 ;	-1.194
	curdlan#FIBCD1	1.295	0.397	0.001	0.518 ;	2.073
	chitin#FIBCD1	0.798	0.397	0.044	0.020 ;	1.575
_cons		-0.009	0.332	0.979	-0.659 ;	0.642
**IL12B**						
Ligand						
	AIF	-2.235	0.257	0.000	-2.739 ;	-1.732
	curdlan	-0.597	0.255	0.019	-1.097 ;	-0.096
	chitin	-0.426	0.255	0.095	-0.927 ;	0.075
Genotype						
	FIBCD1	0.389	0.222	0.079	-0.045 ;	0.824
Interaction						
	AIF#FIBCD1	1.228	0.314	0.000	0.613 ;	1.843
	curdlan#FIBCD1	0.550	0.314	0.080	-0.065 ;	1.165
	chitin#FIBCD1	-0.199	0.314	0.527	-0.814 ;	0.416
_cons		-0.006	0.182	0.976	-0.362 ;	0.351
**IL13**						
Ligand						
	AIF	-0.750	0.558	0.178	-1.843 ;	0.342
	curdlan	-0.010	0.545	0.986	-1.078 ;	1.059
	chitin	0.433	0.545	0.427	-0.636 ;	1.501
Genotype						
	FIBCD1	0.917	0.399	0.022	0.135 ;	1.698
Interaction						
	AIF#FIBCD1	-0.025	0.564	0.965	-1.130 ;	1.080
	curdlan#FIBCD1	0.226	0.564	0.689	-0.879 ;	1.331
	chitin#FIBCD1	-0.872	0.564	0.122	-1.977 ;	0.233
_cons		-0.041	0.394	0.918	-0.813 ;	0.732
**MUC1**						
Ligand						
	AIF	-1.194	0.327	0.000	-1.834 ;	-0.554
	curdlan	0.277	0.306	0.365	-0.323 ;	0.878
	chitin	-0.053	0.306	0.863	-0.653 ;	0.548
Genotype						
	FIBCD1	-1.766	0.148	0.000	-2.055 ;	-1.476
Interaction						
	AIF#FIBCD1	-0.646	0.209	0.002	-1.055 ;	-0.236
	curdlan#FIBCD1	0.268	0.209	0.199	-0.141 ;	0.677

	chitin#FIBCD1	0.071	0.209	0.734	-0.338	;	0.480
_cons		-0.066	0.231	0.776	-0.518	;	0.387
**MUC13**							
Ligand							
	AIF	-0.822	0.588	0.162	-1.975	;	0.331
	curdlan	0.100	0.583	0.864	-1.042	;	1.242
	chitin	-0.096	0.583	0.869	-1.238	;	1.046
Genotype							
	FIBCD1	-3.188	0.484	0.000	-4.137	;	-2.238
Interaction							
	AIF#FIBCD1	-4.313	0.685	0.000	-5.655	;	-2.970
	curdlan#FIBCD1	0.915	0.685	0.181	-0.427	;	2.258
	chitin#FIBCD1	0.098	0.685	0.886	-1.245	;	1.440
_cons		-0.008	0.416	0.986	-0.823	;	0.808
**MUC5AC**							
Ligand							
	AIF	-1.246	1.015	0.220	-3.235	;	0.743
	curdlan	0.262	0.973	0.787	-1.644	;	2.169
	chitin	0.714	0.973	0.463	-1.192	;	2.620
Genotype							
	FIBCD1	-2.143	0.589	0.000	-3.298	;	-0.988
Interaction							
	AIF#FIBCD1	-5.442	0.833	0.000	-7.076	;	-3.809
	curdlan#FIBCD1	0.712	0.833	0.393	-0.922	;	2.345
	chitin#FIBCD1	-0.036	0.833	0.965	-1.669	;	1.597
_cons		-0.052	0.718	0.942	-1.459	;	1.354
**TJP1**							
Ligand							
	AIF	-0.141	0.102	0.168	-0.342	;	0.059
	curdlan	-0.117	0.101	0.244	-0.314	;	0.080
	chitin	-0.143	0.101	0.154	-0.340	;	0.054
Genotype							
	FIBCD1	-0.470	0.078	0.000	-0.623	;	-0.317
Interaction							
	AIF#FIBCD1	-0.234	0.110	0.034	-0.450	;	-0.017
	curdlan#FIBCD1	0.329	0.110	0.003	0.113	;	0.546
	chitin#FIBCD1	-0.005	0.110	0.963	-0.221	;	0.211
_cons		-0.001	0.072	0.991	-0.143	;	0.141
**OCLN**							
Ligand							
	AIF	0.272	0.137	0.048	0.003	;	0.541
	curdlan	-0.739	0.134	0.000	-1.002	;	-0.475
	chitin	-0.577	0.134	0.000	-0.840	;	-0.313
Genotype							

	FIBCD1	1.037	0.099	0.000	0.844	;	1.231
Interaction							
	AIF#FIBCD1	-0.585	0.140	0.000	-0.859	;	-0.311
	curdlan#FIBCD1	0.510	0.140	0.000	0.236	;	0.784
	chitin#FIBCD1	0.297	0.140	0.033	0.023	;	0.571
_cons		-0.018	0.097	0.851	-0.209	;	0.172
**ICAM1**							
Ligand							
	AIF	0.115	0.408	0.777	-0.069	;	0.916
	curdlan	3.832	0.408	0.000	3.031	;	4.632
	chitin	0.408	0.408	0.318	-0.392	;	1.208
Genotype							
	FIBCD1	4.283	0.408	0.000	3.483	;	5.083
Interaction							
	AIF#FIBCD1	-0.173	0.557	0.765	-1.305	;	0.959
	curdlan#FIBCD1	-3.679	0.557	0.000	-4.810	;	-2.547
	chitin#FIBCD1	-0.393	0.557	0.496	-1.525	;	0.738
_cons		-0.077	0.289	0.791	-0.642	;	0.489
