# Supplementary material for: FIBCD1 Binds Aspergillus fumigatus and Regulates Lung Epithelial Response to Cell Wall Components
Source: Front Immunol. 2018 Sep 18;9:1967. doi: 10.3389/fimmu.2018.01967 (PMC6153955; doi:10.3389/fimmu.2018.01967)
Supplement: Supplementary file 7 [file Data_Sheet_2.docx]

# Supporting information

## Silver staining

After SDS-PAGE, the gel was incubated in 50 % ethanol/10 % acetic acid for 10 min, in 5 % ethanol/7 % acetic acid for 10 min, washed in deionized water (four times quick, once for 5 min), incubated in 100 mL 0.06 mM DTT for 15 min, washed again, and incubated in 100 mL 0.1 % (w/v) AgNO_3_. The solution was discarded and the gel washed with a couple mL of 3 % Na_2_CO_3_/0.0185 % formaldehyde until the solution turned black. The solution was discarded and the gel was developed by incubating with 150 mL 3 % Na_2_CO_3_/0.0185 % formaldehyde until the desired coloration was obtained and the reaction stopped by incubating in 2.4 % citric acid for 15 min.

## Cell lysates

Cell lysates were prepared by washing the cells of a confluent culture twice with DPBS and incubating with 28 µL/cm^2^ TBS/1 % Triton X-100/protease inhibitors (1 tablet per 50 mL) at 4°C for 30 min. Cellular debris were sedimented by centrifugation at 4°C, 10,000 x g for 10 min and supernatants were analyzed by SDS-PAGE using equal amount of cell lysate. Unreduced and reduced cell lysates were prepared by boiling three parts cell lysate with one part SDS-PAGE sample buffer supplemented with one tenth 0.02 M IAA and 0.6 M DTT, respectively, on a 99°C heating block for 1 min. The samples were alkylated by adding one tenth 1.4 M IAA, separated on a 4-12 % polyacrylamide gradient gel using MOPS running buffer and analyzed by Western blotting as described in the following section.

## Stimulation of wild type A549 cells

For stimulation of wild type A549 cells, cells were harvested by incubation with 0.5 % trypsin/EDTA in DPBS and centrifugation at 350 x g for 5 min. Cells were suspended in media, counted using a haemocytometer, and seeded at a density of 3·10^5^ cells/well/2 mL media in 12-well culture plates (75% confluence). After approximately 8 hours, the adherent cells were washed with sterile DPBS, added 2 mL serum-free media (RPMI media supplemented with 2mM L-glutamine, 50U/mL penicillin, and 50µg/mL streptomycin), and incubated overnight. Then, cells were stimulated by removing the media and adding 2 mL of fresh serum-free media containing 5 – 25 µL stimulant diluted in sterile DPBS to each well and incubating 0, 4, 8, and 12 hours. At each time point, culture supernatant was removed from the cells and used for detection of secreted IL-8 by use of the human CXCL8/IL-8 DuoSet kit and keeping to the recommendations of the manufacturer.
